# Leaf Mass per Area (LMA) and Its Relationship with Leaf Structure and Anatomy in 34 Mediterranean Woody Species along a Water Availability Gradient

**DOI:** 10.1371/journal.pone.0148788

**Published:** 2016-02-11

**Authors:** Enrique G. de la Riva, Manuel Olmo, Hendrik Poorter, José Luis Ubera, Rafael Villar

**Affiliations:** 1 Area de Ecología, Facultad de Ciencias, Universidad de Córdoba, 14071 Córdoba, Spain; 2 Plant Sciences (IBG-2), Forschungszentrum Jülich GmbH, 52425 Jülich, Germany; 3 Area de Botánica, Facultad de Ciencias, Universidad de Córdoba, 14071 Córdoba, Spain; Estacion Experimental de Zonas Aridas - CSIC, SPAIN

## Abstract

Leaf mass per area (LMA) is a morphological trait widely used as a good indicator of plant functioning (i.e. photosynthetic and respiratory rates, chemical composition, resistance to herbivory, etc.). The LMA can be broken down into the leaf density (LD) and leaf volume to area ratio (LVA or thickness), which in turn are determined by anatomical tissues and chemical composition. The aim of this study is to understand the anatomical and chemical characteristics related to LMA variation in species growing in the field along a water availability gradient. We determined LMA and its components (LD, LVA and anatomical tissues) for 34 Mediterranean (20 evergreen and 14 deciduous) woody species. Variation in LMA was due to variation in both LD and LVA. For both deciduous and evergreen species LVA variation was strongly and positively related with mesophyll volume per area (VA or thickness), but for evergreen species positive relationships of LVA with the VA of epidermis, vascular plus sclerenchyma tissues and air spaces were found as well. The leaf carbon concentration was positively related with mesophyll VA in deciduous species, and with VA of vascular plus sclerenchymatic tissues in evergreens. Species occurring at the sites with lower water availability were generally characterised by a high LMA and LD.

## Introduction

Plant traits can determine species differences in productivity and performance and therefore the distribution of species in nature [1–5]. In this regard, leaf traits are fundamental for ecosystem functioning, being related with important processes such as carbon gain or litter decomposability [1, 4, 6, 7]. One of the central variables among the leaf traits is the leaf mass per area (LMA), which is the ratio between leaf dry mass and leaf area [6, 8]. In spite of being a morphological trait, LMA (or its inverse, specific leaf area, SLA) is highly correlated with leaf processes such as maximum photosynthetic rate [1, 9, 10, 11], whole-plant activities such as the species’ potential growth rate [12–15] and ecosystem processes such as decomposition rate [7, 16, 17]. The LMA of a species is therefore a good indicator of the position of that species along an axis based on resources acquisition (Leaf Economic Spectrum) [1]. However, despite its physiological and ecological relevance, the traits which underlie the interspecific variation in LMA are still poorly understood [6, 18].

In its simplest form, LMA can be broken down into the product of leaf density (LD, g mL^−1^) and the leaf volume to area ratio (LVA, mL m^-2^, also called leaf thickness) [8, 19]:

$$LMA\;(g\;m^{-2})\;=\;LVA\;(mL\;m^{-2})\;\times \;LD\;(g\;mL^{-1})$$

The relative importance of the two variables in explaining variation in LMA is not consistent. Thus, while some studies [20–23] found that LD was the main component that differed between low- and high-LMA species, another study [24] found that variation in LMA was mainly due to variation in LVA. Villar et al. [18] and Niinemets [25] found that LMA variation depended equally on LVA and LD, but the class difference between deciduous and evergreen species was mainly determined by LVA, whereas variation within each group was largely due to density. Also, the relationships between LVA and LD are variable; they have been reported to be negatively correlated [26], but they may also vary independently [22]. These contradictory results suggest that the causes of LMA variation do not necessarily follow a global pattern, and depend on the nature of the species compared and their environment [6, 22, 27]. In order to obtain better insight into this variability, we need more studies testing the relationship between LMA and its two components (LVA and LD), especially for plants growing under natural conditions with diverse species pools in different environments.

The second step towards understanding LMA variation is to consider the different anatomical tissues that shape the leaves. Both LVA and LD are determined by the composition of the different anatomical tissues: epidermis, mesophyll and vascular plus sclerenchymatic tissue, as well as air spaces. The LVA is the sum of the volumes of all the tissues per unit leaf area (VA) plus the air spaces [6, 18]. The VA of each tissue is often described as “tissue thickness” (μm), but as this is technically incorrect for non-laminar tissues such as sclerenchyma, or for air spaces, we will use VA throughout this paper. The other component, LD, is the sum of the densities of each tissue, weighted by the volumetric fraction of that tissue [6, 18]. The various anatomical tissues generally have different physiological functions: mesophyll is related to photosynthesis and transpiration [28] and air spaces can potentially determine gas exchange variables [29]. So, different combinations of the number and layering of these tissues will have different physiological consequences. Differences in the anatomical composition of the leaves would then affect LVA and LD, and consequently leaf functioning.

Another consequence of variation in the composition of the anatomical tissues is that it may also affect the chemical composition; for example, a high presence of vascular and sclerenchymatic tissue may cause a high C concentration in the leaf, whereas a large presence of mesophyll may increase the concentration of N [6, 20, 30, 31]. Both C and N concentration are strongly related to plant functioning as well [1, 4, 5, 6].

To understand the functional consequences of variation in plant traits or anatomical components, we need more insight into the performance of plants under different environmental conditions [8]. Thus, for example, in habitats with high irradiance and low water availability, such as Mediterranean environments, most species have sclerophyllous evergreen leaves with high LMA, low nutrient concentrations and low maximum photosynthetic rate [10–12, 32–34]. Light and water therefore exert an important effect, selecting those species capable of growing and reproducing under such environmental conditions. In relation to this, several studies have found that species with high LMA often occur in areas with low rainfall and high temperature and radiation [1, 34, 35]. However, how LVA and LD or leaf anatomy explain LMA variation remains largely unexplored along natural environmental gradients (but see Witkowski and Lamont [8] and Niinemets [34]).

In this study, we explored the variation in LMA and its components (both morphological and anatomical) for 34 Mediterranean (20 evergreen and 14 deciduous) woody species growing along a local environmental gradient, which differed mainly in water availability (see de la Riva et al. [36]). We asked the following questions:

1. To what extent do LVA and LD explain the interspecific variation in LMA?
2. How does variation in the different leaf anatomical tissues explain the differences in LVA, LD and LMA?
3. Are the differences in chemical composition (C and N concentrations) related to the leaf anatomy and morphology (LMA, LVA and LD)?
4. What influence does the soil water gradient exert on leaf traits (structural and anatomical)?

We analysed these questions at two levels: a) considering all the species together and b) at the level of functional groups (within deciduous species or within evergreens). With this approach, we wanted to find general or particular patterns, depending on the group of species considered.

## Materials and Methods

### Site characterisation and species selection

The study was conducted in a Mediterranean habitat with forests and shrublands, located in the Sierra Morena mountains, in the south of Spain. The area is characterised by a continental-Mediterranean climate with cold, wet winters and dry, warm summers. The mean annual temperature is 17.6°C and the mean annual precipitation is 536 mm, of which 94% falls from October to June [data from AEMET (Agencia Estatal de Meteorología, Spain) for the 1971–2000 period]. The bedrock is formed by a siliceous substratum, which produces neutral or slightly acid soils (Leptosols). Several shrub species—such as *Cistus albidus*, *Quercus coccifera* and *Rosmarinus officinalis*—are abundant at exposed sites on drier and shallow soils, while broadleaf deciduous trees—such as *Alnus glutinosa*, *Celtis australis*, *Fraxinus angustifolia* and *Ulmus minor—*are dominant at wetter sites with deeper soils (see S1 Table). Nine sampling sites were selected along the environmental gradient, from ridges to valley bottoms, with the aim of spanning a broad range of soil resource availabilities, mainly for water but also for nutrients. There was no climatic difference across the gradient (see de la Riva et al. [36] for more details) and the differences in water availability were due not to differences in annual rainfall but to the topography (S2 Table). For ease of reference, we will refer to this as a water gradient in most of the text. For trait measurements (see details below), we selected the most abundant woody species (see de la Riva et al. [36], S1 Table). This made a total of 34 selected species (20 evergreen and 14 deciduous species). In late spring (June 2012), during the peak of plant growth, six healthy adults of the most dominant woody plant species were randomly selected for measurement of leaf traits. A few species could be found at several sites, but most were found at only one or two. As our aim was not to analyse the intraspecific variation in leaf traits, we only selected one site for each species.

### Leaf measurements

Leaf samples were collected mainly in private orchards, with the permission of the land owners. The field studies did not involve any endangered or protected species. A few branches with young, fully expanded leaves were collected from each selected individual plant. These branches were stored in plastic bags to prevent water loss and were transported to the laboratory, where they were maintained for 24 h in darkness with the basal portion of their stem submerged in water at 10°C to allow complete re-hydration [37, 38]. Subsequently, a subsample of the leaves was scanned and dried in an oven at 70°C for at least 48 h, after which they were weighed to obtain their dry mass. Leaf area was calculated with the Image Pro-Plus v4.5 software (Media Cybernetics, MD, USA). The leaves were then ground with a stainless steel mill, and the N and C concentrations measured using an elemental analyser (Eurovector EA 3000; EuroVector SpA, Milan, Italy).

Another subsample was used for anatomical analysis. One healthy leaf of three individuals (from those selected for LMA measurements) per species was selected. From the middle part of the leaves, 5-mm-wide pieces were taken and directly fixed in formaldehyde-acetic acid (FAA; 35–40% formaldehyde, 70% ethanol and 100% acetic acid, 1:8:1 v/v). The leaf sections were dehydrated with a series of progressively increasing ethanol concentrations, starting at 50% and ending at 100%. They were then embedded using a JB-4 embedding kit (Polysciences Ltd., Warrington, PA, USA); subsequently, 3-μm-thick slices were cut by a microtome (Leica Reichert-Jung Autocut 2055 microtome, Wetzlar, Germany) and these were stained with Toluidine blue (5%). Photographic images were taken via a light microscope, using a Nikon D700 camera. The total area of each cross-section was determined using the image analysis procedures of Adobe Photoshop CS3 (Adobe Systems Inc., San Jose, CA). Two subsamples for each cross-section and individual were selected for measurement of the area occupied by the upper and lower epidermis, the palisade and spongy parenchyma, the vascular and sclerenchymatic tissue and the air spaces (see S1 Fig for the cross-sections of the 34 species). We also measured the leaf thickness in 10 random places for each cross-section of the leaf.

The mean values of all the anatomical measurements were calculated per leaf, and subsequently averaged for each species. The mean values of each tissue were expressed in two ways: the absolute volume of each tissue per unit leaf area (VA, also denoted as the thickness of each tissue) and the volume fraction (percentage of the leaf section occupied by the different tissues and air spaces). Most studies have only determined the volume fractions, but we decided to use both parameters as they answer different questions. The absolute values are employed in order to determine the contribution of each tissue to the LVA (leaf thickness), whereas the volume fractions are analysed in relation to LD. For simplicity in the presentation of the results, we consider: 1) epidermis (upper and lower epidermis), 2) mesophyll (palisade and spongy parenchyma), 3) vascular and sclerenchymatic tissue and 4) the air spaces.

### Soil water measurements

In May 2012, for each site, eight soil samples were taken using an auger, to a maximum depth of one meter. The soil water content was quantified by the gravimetric method, weighing the soil samples when fresh and after oven-drying at 100°C for 48 h. From these measurements, we calculated an integrative variable of the whole soil profile for each sample (soil water content, SWC) as: (fresh soil mass—dry soil mass)/ area of the auger section (5 cm^2^). The soil water content (L m^-2^) for each sample site was the average of these eight samples.

### Data and statistical analysis

The LMA was calculated as the ratio of leaf dry mass and leaf area. The LD was calculated as the ratio of LMA and LVA (leaf thickness) [8]. The relationships among morphological (LMA, LVA and LD) and anatomical (epidermis, mesophyll, vascular plus sclerenchyma and air spaces) traits were determined by linear regressions, similar to Castro-Díez et al. [22]. However, in these regressions one fraction of the explained variability results from the covariance between these components [39]. To avoid this covariation, we used the method based on the Sum of Squares (SS) decomposition from Lepš et al. [40]. The SS can be decomposed into the amount of variability explained by individual terms of the model and the unexplained variability (error). Thus, SS_total_ = SS_factor 1_+ SS_factor 2_ + SS_factor 1 × factor 2_ + SS_error_. If the two effects are positively correlated (i.e. LD and LVA), then the SS_total_ will be higher than when the two effects are independent. To obtain the variability explained by each element without covariation, we considered, for example for factor 1, that SS_factor 1_ = SS_total_−SS_error_—SS_factor 2_—SS_factor 1 × factor 2_. To obtain the variability explained by each factor we weighted the results for the total variability explained by the model, then, for factor 1 for example, Factor var 1 (%) = 100 × [SS_factor 1_/SS_total_]. The Lepš method was developed for a factorial ANOVA, so some differences must be considered for linear regression models (see S1 Appendix).

The relationships of the morphological traits with both the anatomical traits and the C and N concentration were also explored by linear regressions. All the relationships were analysed in three ways: considering all species together, or deciduous and evergreen species separately.

In order to allow for the influence of species evolutionary history, Pearson correlation analyses were carried out for all the above-described relationships, by fitting a phylogenetic generalised least squares model. By calculating phylogenetically independent contrasts (PIC), we can assess the impact of phylogeny on our results [41, 42]. For these PICs, we used the pgls function of the caper package [43] for R (R Development Core Team 2011), which addresses phylogenetic non-independence among species by incorporating covariance between taxa into the calculation of the estimated coefficients. The phylogenetic relationships between species (see S2 Fig) were obtained with the help of the Phylomatic program, as implemented in Phylocom 4.2, and the reference phylogeny contained in R20120829.new [41]. We resolved the topology of the tree (below the family level) with information from published phylogenies [42, 44, 45]. The age estimates for nodes in the tree were taken from Verdú et al. [42] and branch lengths were adjusted by using the BLADJ algorithm in Phylocom 4.2. In addition, we conducted a deciduous vs. evergreen comparison for morphological and anatomical traits (in both cases: absolute and percentage values) by fitting a phylogenetic generalised least squares model (PGLS), using the pgls function of the caper package [43] for R.

To summarise all the information, we calculated a correlation network (see Poorter et al. [46]) based on the overall correlations (Pearson’s correlation analysis) between the morphological, anatomical and chemical leaf traits. The network analysis was carried out for all the species together and for the deciduous and evergreen species separately.

In order to investigate the relationship between the soil water content and the leaf structural and anatomical traits, we carried out a simple regression analysis (R Development Core Team 2011) with all the species, or with the deciduous and evergreen species separately.

## Results

### Leaf morphology (LMA, LVA and LD) and anatomy

**Fig 1:**
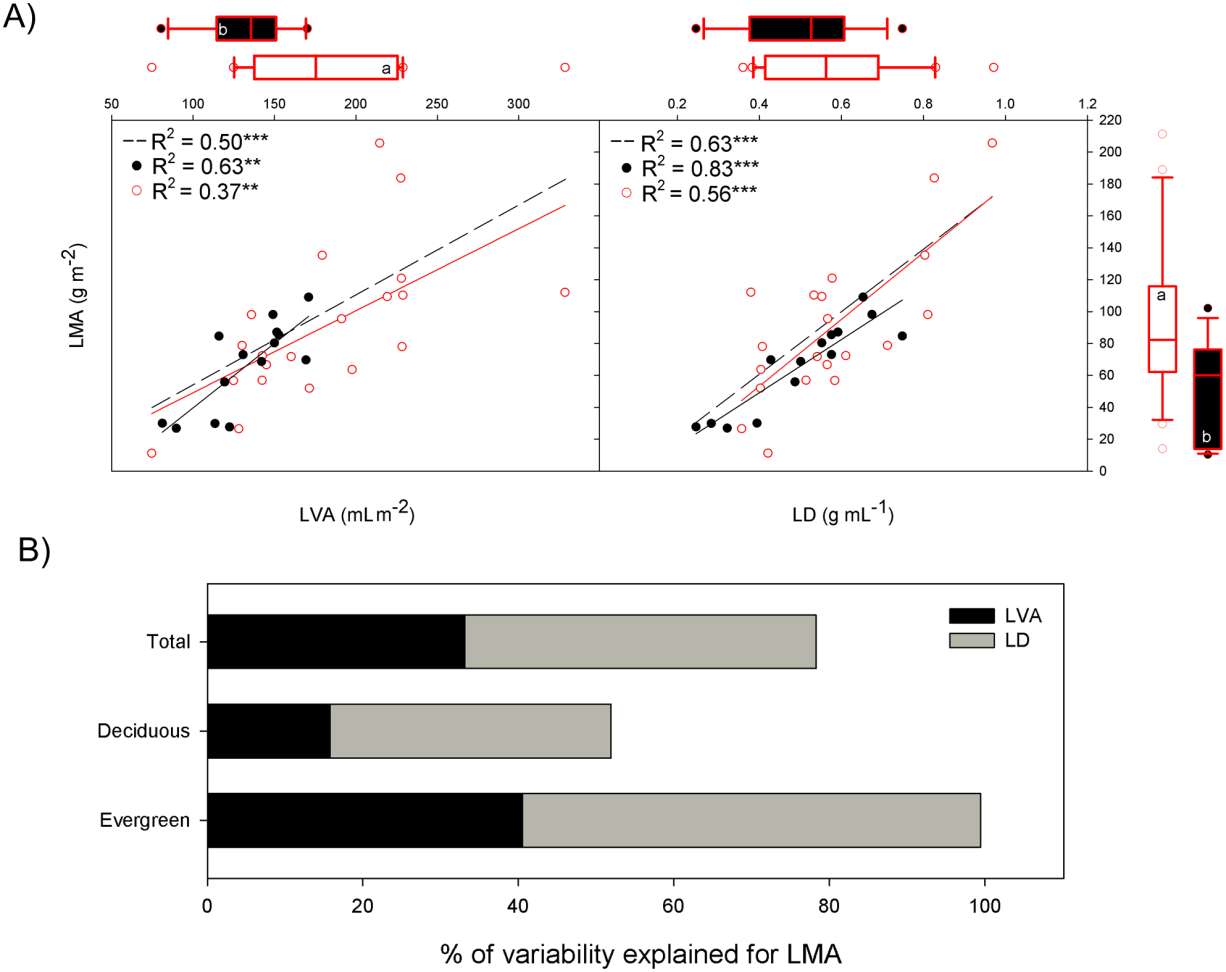
**A)** Linear regressions of leaf mass per area (LMA) with leaf volume per area (LVA or thickness) and leaf density (LD), for deciduous species (dark line and circles), evergreens (red line and empty red circles) and all the species (dashed line). The level of significance is expressed as follows: * *P* < 0.05, ** *P* < 0.01, *** *P* < 0.001. Box plots (median and 1^st^ and 3^rd^ quartiles) of deciduous vs. evergreen species are included in the margins; the significant results are based on the phylogenetic generalised least squares model (PGLS). The results based on PGLS can be found in S3 Table, Supporting Information. Whiskers show the minimum and maximum values that fall within 1.5x the length of the box away from the interquartile range; data further away are shown as outliers. **B**) Decomposition of the total variability explained by LVA and LD.

Considered over all the species, LMA was positively related with both leaf thickness (LVA; R^2^ = 0.50) and leaf density (LD; R^2^ = 0.63) (Fig 1A). There was no relationship between LD and LVA (*P* > 0.05, data not shown). These patterns were also found for the deciduous and evergreen species considered independently (Fig 1A). Evergreen species showed higher values of LMA and LVA than deciduous species (Fig 1A), but no overall differences were found in LD (Fig 1A, S3 Table). The partitioning of the total variability of LMA among the morphological traits considered (without covariations) demonstrated that 45% of the LMA variation was due to LD and 33% to LVA (Fig 1B). Similarly to when considering all the data, LMA variation within each functional group was mainly due to variation in LD (Fig 1B). However, the variation in LMA was better explained within evergreens (59% LD and 40% LVA) than within deciduous species (36% LD and 16% LVA).

**Fig 2:**
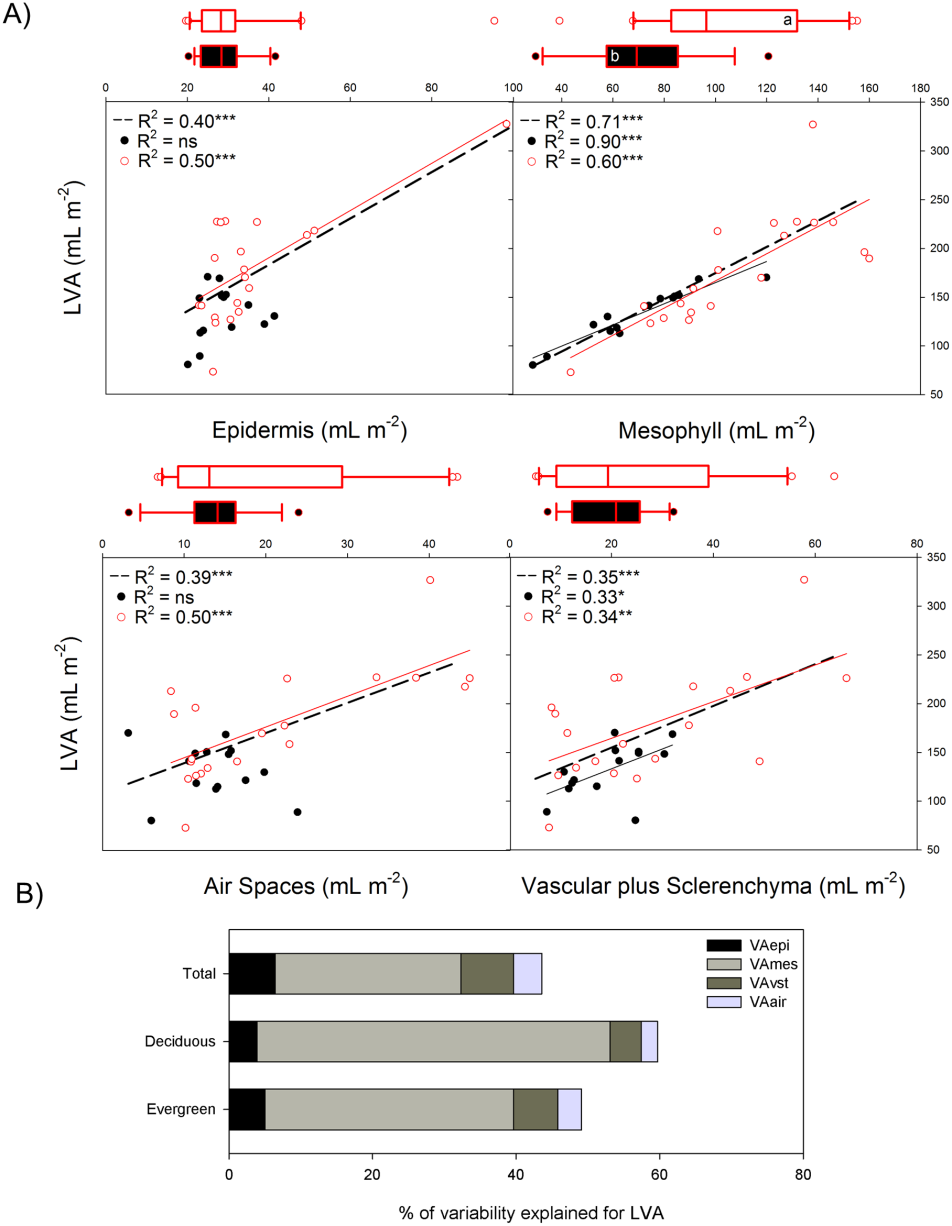
**A)** Linear regressions of leaf volume per area (LVA or thickness) with anatomical tissues, for deciduous species (dark line and circles), evergreens (red line and empty red circles) and all the species (dashed line). In brackets the slopes of the regression lines are given. The level of significance is expressed as follows: * *P* < 0.05, ** *P* < 0.01, *** *P* < 0.001. Box plots (median and 1^st^ and 3^rd^ quartiles) of deciduous vs. evergreen species are included in the margins; the significant results are based on the phylogenetic generalised least squares model (PGLS). The results based on PGLS can be found in S3 Table, Supporting Information. Whiskers show the minimum and maximum values that fall within 1.5x the length of the box away from the interquartile range; data further away are shown as outliers. **B)** Decomposition of the total variability explained by anatomical tissues, without covariations.

The overall variation in LMA, especially in the LVA, was explained partially by the different tissues. Considering all species, the increase in LVA was caused by an increase in the volume per area of all the tissues, but predominantly by the increase in the mesophyll (R^2^ = 0.71) (Fig 2A). Although, in the case of air spaces, the relationship with LVA disappeared when the phylogenetic relationships were taken into account (S4 Table).

The relationship between LVA and the anatomical composition depended on the functional group (deciduous vs. evergreen). For deciduous species, LVA was only related to mesophyll VA (thickness) (R^2^ = 0.90) and vascular plus sclerenchyma VA (R^2^ = 0.33) (Fig 2A), while for evergreen species the increase in LVA was due to an increase in all the tissues, though it was better explained by mesophyll VA (R^2^ = 0.60) than by the other tissues. The partitioning of the variability showed the importance of the mesophyll VA in the explanation of leaf thickness (Fig 2B) and the secondary role of the other tissues (which together explained less than 20% of the variance, for both groups of species).

With regard to the relationships of LD with the proportions of anatomical tissues, we only found some marginally significant results. Thus, LD was related negatively to the proportion of epidermis and marginally with air spaces (0.05 < *P* < 0.10) (S5 Table). Considering each functional group separately, LD was only marginally and positively related to mesophyll for the deciduous species (0.05 < *P* < 0.10) and to vascular plus sclerenchymatic tissue for the evergreens (0.05 < *P* < 0.10) (S5 Table).

The evergreens differed from the deciduous species only in mesophyll VA, which was higher in evergreens (*P* < 0.001, Fig 2A). Considering the volumetric fractions, the evergreens showed a higher mesophyll and lower epidermis fraction (*P* < 0.05) than the deciduous species (S2 Table). No systematic differences between the deciduous and evergreen species were found for any of the other tissues.

### Relationships of leaf morphology and anatomy with chemical composition

**Fig 3:**
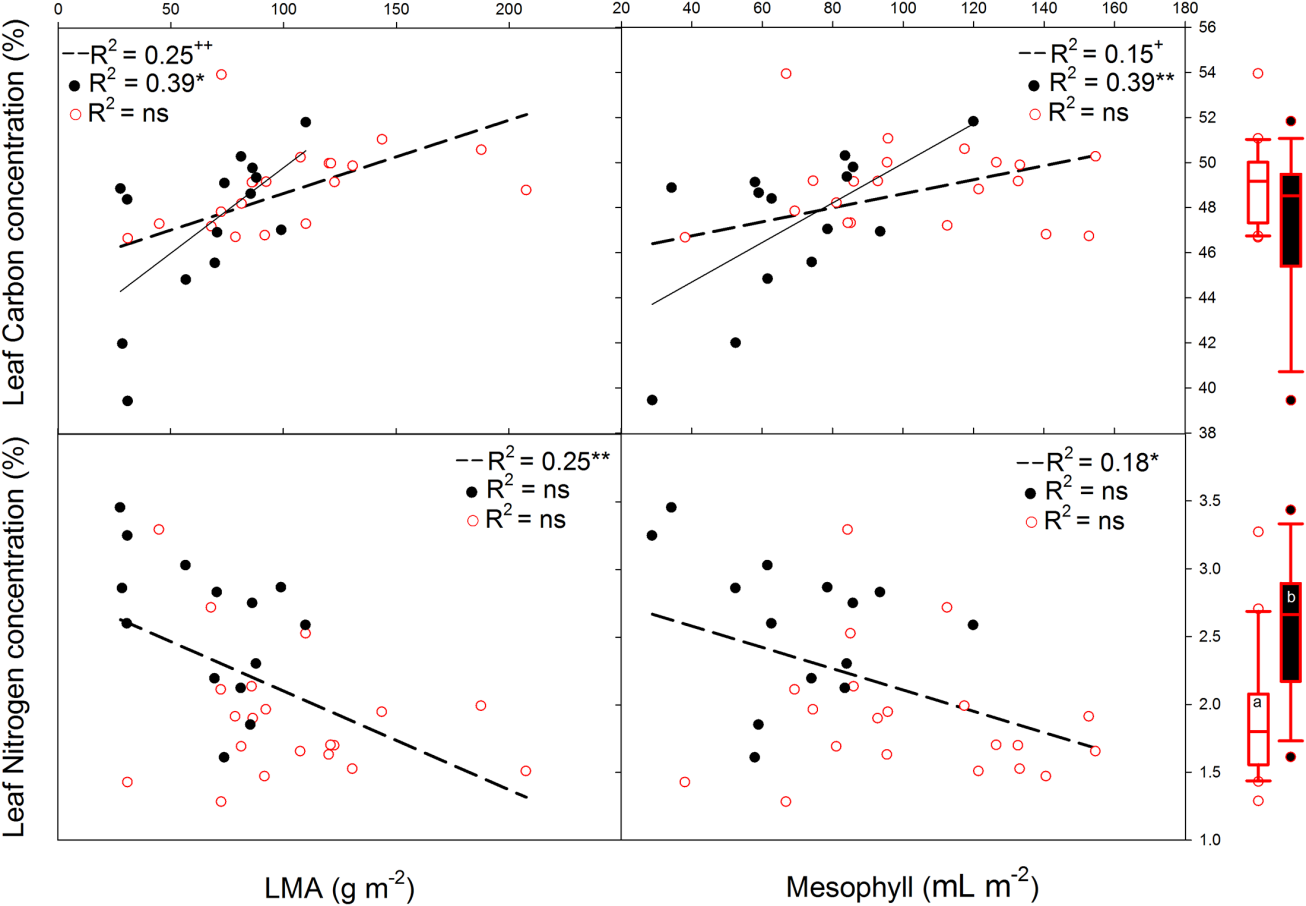
**A)** Linear regressions of leaf mass area (LMA) and mesophyll with leaf carbon concentration (LCC) and leaf nitrogen concentration (LNC), for deciduous species (dark line and circles), evergreens (red line and empty red circles) and all the species (dashed line). In brackets the slopes of the regression lines are given. The level of significance is expressed as follows: * *P* < 0.05, ** *P* < 0.01, *** *P* < 0.001, ^+^ is shown when the relationship is phylogenetically dependent. Box plots (median and 1^st^ and 3^rd^ quartiles) of deciduous vs. evergreen species are included in the margins; the significant results are based on the phylogenetic generalised least squares model (PGLS). The results based on PGLS can be found in S3 Table, Supporting Information. Whiskers show the minimum and maximum values that fall within 1.5x the length of the box away from the interquartile range; data further away are shown as outliers.

The relationships of the C and N concentrations with the morphological and anatomical traits differed strongly (Fig 3 and Table 1). Leaf C was positively correlated with LMA, LD and LVA. However, all the correlations (across the 34 species) of leaf C with the morphological and anatomical traits were influenced by taxonomy; no significant results were found when including PIC in the pairwise correlations (except for LVA and vascular plus sclerenchymatic tissue, *P* < 0.05; S4 Table).

**Table 1:** Linear regressions of the morphological and anatomical traits with leaf carbon concentration (LCC) and leaf nitrogen concentration (LNC). The positive or negative relations (Rel) and the R^2^ of the regressions are shown.

	LCC						LNC					
	All		Deciduous		Evergreen		All		Deciduous		Evergreen	
	Rel	R²	Rel	R²	Rel	R²	Rel	R²	Rel	R²	Rel	R²
LMA	+	0.25 **	+	0.39 *	ns		-	0.25 **	ns		ns	
LVA	+	0.14 *	+	0.30 *	ns		-	0.22 **	ns		ns	
LD	+	0.19 **	ns		ns		-	0.13 *	-	0.30 *	ns	
Epidermis VA	ns		ns		ns		ns		ns		ns	
Mesophyll VA	+	0.15 *	+	0.39 **	ns		-	0.18 *	ns		ns	
Vas+Scl VA	+	0.12 *	ns		+	0.38 **	ns		ns		ns	
Air spaces VA	ns		ns		ns		ns		ns		ns	

The level of significance is expressed as follows:

* *P* < 0.05,

** *P* < 0.01.

The groups of evergreen and deciduous species did not differ in their C concentrations (S2 Table). Within the deciduous species a higher LMA was related to a higher C concentration (Fig 3). This increase in C concentration was related to higher values of mesophyll thickness (R^2^ = 0.39, *P* < 0.05, Fig 3). In the case of the evergreen species, the increase in C concentration was related to an increase in the VA of vascular plus sclerenchymatic tissues (R^2^ = 0.38, *P* < 0.01, Table 1).

Leaf N concentration showed negative relationships with all morphological traits (LMA, LVA and LD) and mesophyll thickness (Fig 3 and Table 1). Deciduous species showed a higher N concentration than evergreen species (*P* = 0.001; S2 Table). Within each functional group, only the LD of the deciduous species was negatively related with N concentration (Table 1).

### Network-correlations

**Fig 4:**
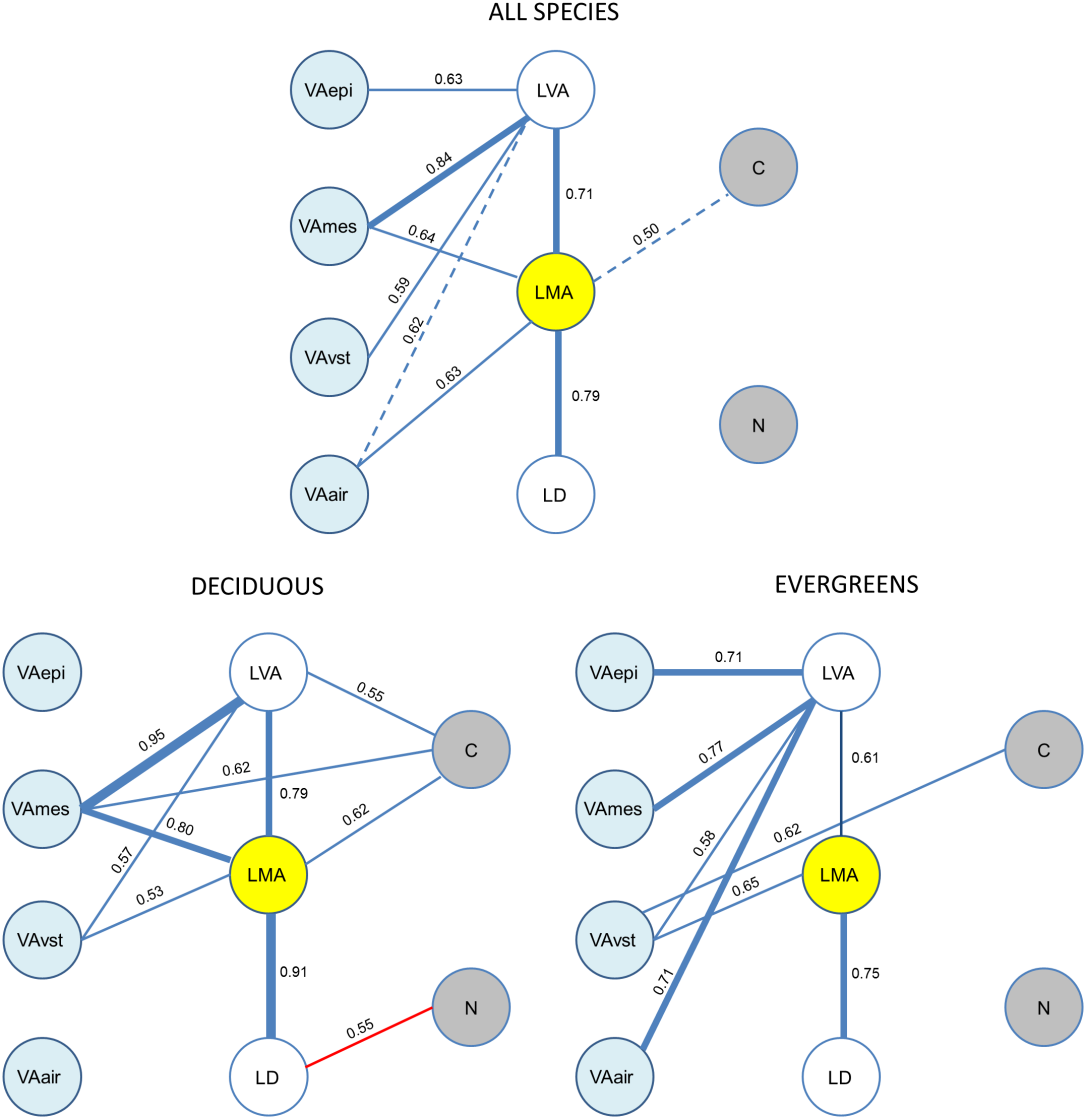
Correlation network for morphological (white), anatomical (blue) and chemical traits (grey), describing the interrelations with LMA (yellow). Blue lines indicate positive correlations and red lines negative correlations. Dashed lines indicate phylogenetic independence. The correlation coefficient is shown. Thin lines, 0.5<|r|<0.707 (0.25<R^2^<0.50); intermediate lines, 0.707<|r|< 0.866 (0.50<R^2^<0.75); bold lines, |r|>0.866 (R^2^>0.75).

As a summary we present here all the correlations in a network map, for all species and within the evergreen or deciduous species (Fig 4). For all three groups considered (all species, evergreens or deciduous), the variation in LMA was due to variation in both LVA and LD. However, the causes of the variation in LVA differed between the deciduous and evergreen species. For the deciduous species, LVA was mostly explained by mesophyll VA, whereas for the evergreens the variation in LVA was explained by all tissues. There were few significant correlations between leaf composition (C and N) and LVA or the anatomical traits. Nevertheless, leaf C seemed to be related to LMA (at least in some groups) and leaf N to LD, but only for the deciduous species.

### Relationships of leaf morphology and anatomy with environmental factors

**Fig 5:**
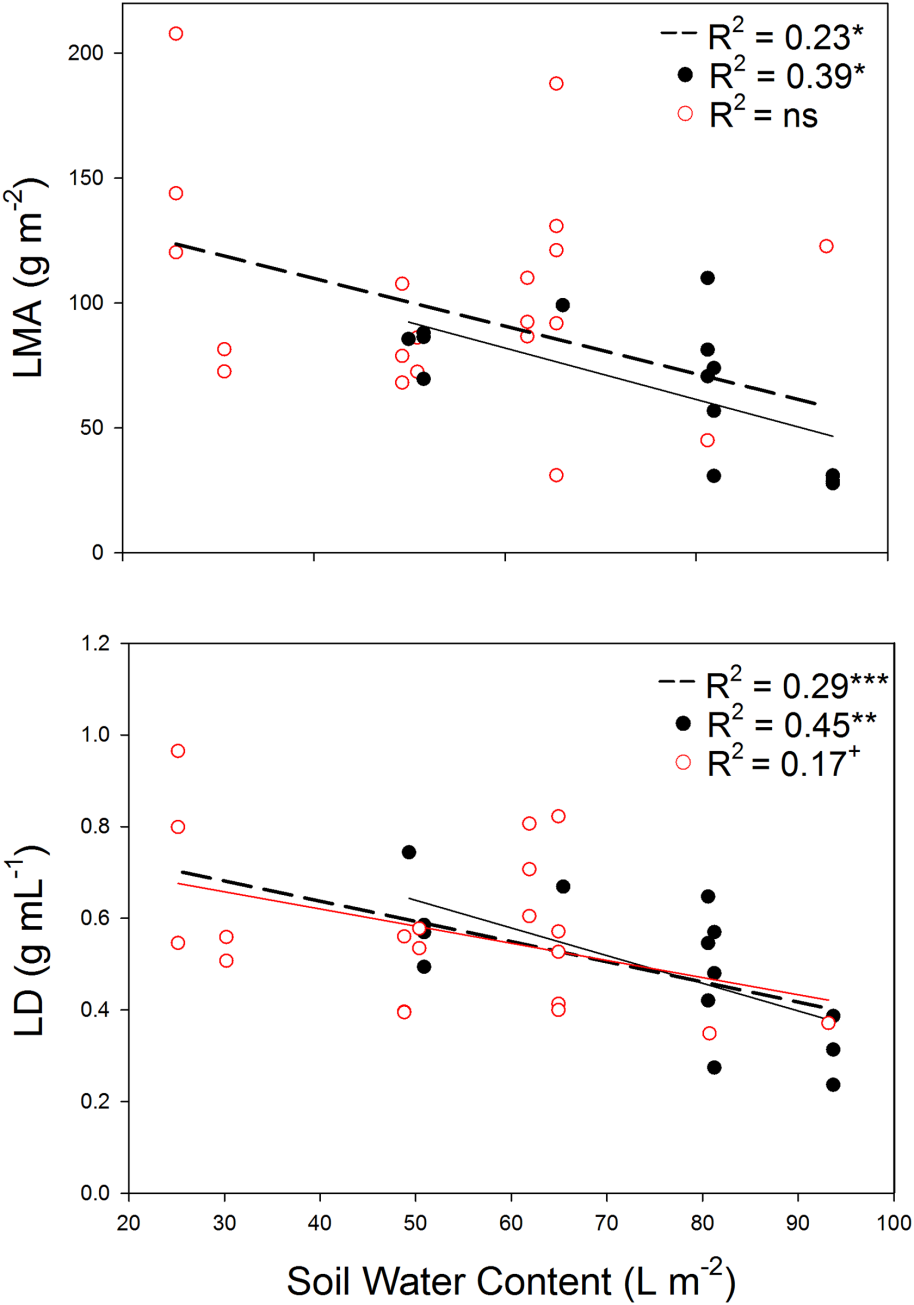
Linear regressions between soil water content and morphological traits (leaf mass per area, LMA and leaf density, LD) for deciduous species (dark line and circles), evergreens (red line and empty red circles) and all the species (dashed line). The level of significance is expressed as follows: ^+^ 0.05 < *P* < 0.10, ** *P* < 0.01, *** *P* < 0.001.

Both LMA and LD were negatively correlated with soil water content (*P* < 0.01, Fig 5). So, species with high LMA and LD were found in habitats with low water availability. In addition, similar results were found for LD and soil water content when deciduous (*P* < 0.05) and evergreen species (0.05 < *P* < 0.10) were considered separately. No relationships were found between LVA or the anatomical traits and soil water content (data not shown), in any case.

## Discussion

We found large variability in the values of leaf morphological traits, anatomical tissues and elemental chemical composition among the 34 woody species studied. For instance, according to Poorter et al. [6] the LMA values among terrestrial species in the field generally ranges from 30 to 330 g m^−2^ (based on the 5th and 95th percentiles of the overall distribution of data). In our study, LMA values varied between 27 to 207 g m^−2^, thus covering a large part of the variation in LMA among woody angiosperms. Our results clearly show that this LMA variation was strongly correlated with variation in both the related morphological traits (LVA and LD), through differences in anatomical composition. The species with high LMA and LD were found in habitats with low water availability. We discuss these results in more detail below.

### Relationships among LMA, LVA and LD

The LMA showed stronger dependency on LD (45%) than on LVA (33%), but this difference was very small (Fig 1B). Hence, these results support previous field studies with woody species [25] and herbaceous species [47], where variation in LMA was found to depend equally on variation in LVA and LD. However, they contrast with those for seedlings of woody species growing in controlled conditions, for which Castro-Díez et al. [22] and Villar et al. [18] reported strong coordination of LVA and LMA as the result of deciduous vs. evergreen differences. These contradictory results suggest that the relationships of LMA with LVA and LD can present large differences depending on the group of species (evergreens, deciduous) [18], age [48] and environmental conditions [8, 27]. In addition, we found no relationship between LVA and LD, indicating that their across-species variation is the result of different mechanisms [25].

Our results show that the deciduous species had lower LVA values than the evergreens, with no differences between leaf habits for LD, similar to the findings of Villar et al. [18]. However, contrary to that study, both groups maintained similar patterns, with positive relationships of LMA with LD and LVA. This similar pattern between different leaf habits suggests that a certain degree of convergence with leaf structure exists. So, the increase of LMA as a result of higher values of LD and LVA seems to be a common pattern for Mediterranean woody species growing in field conditions, independent of leaf habit.

### Relationships of anatomical structure with LVA and LD

In accordance with previous studies [18, 22, 24, 30, 49], our results show that, in general, variation in LVA is best explained by variation in the mesophyll VA, especially for deciduous species (Fig 2B). This could be explained by changes in the number of mesophyll cell layers and/or in cell size [18, 34, 50].

Nevertheless, some modest differences depended on leaf habit: the LVA of deciduous species depended strongly on variation in mesophyll VA (Fig 2), while for evergreen species LVA increased not only with mesophyll VA, but also to some extent with the epidermal VA and vascular plus sclerenchymatic tissues and air spaces VA. For deciduous species, the strong relationship between mesophyll VA and LVA could be related to their high rates of photosynthesis per unit leaf mass, which may allow them to be more competitive during the favourable season [51]. In sclerophyllous evergreen leaves, the high LMA and LVA were also due to the structural tissues (vascular and sclerenchymatic tissues VA) [8], which can confer higher leaf resistance to water diffusion from the vein to the mesophyll [22]. Also, evergreen leaves have more air spaces, which can facilitate CO_2_ diffusion towards the mesophyll [34]. Other studies have also found that variation in LMA depends on variation in vascular and sclerenchymatic tissues [20, 21, 52]. Evergreen leaves often have greater LVA and allocation to mechanical tissue, which could avoid irreparable damage as the result of frost or drought during unfavourable conditions [53].

Similar to Villar et al. [18], we found no difference in leaf density between deciduous and evergreen species. To understand this variation from the results for anatomy, we have considered the fractional volumes of the tissues rather than their volume of tissue per area (VA or thickness) [18]. The volumetric fractions of the mesophyll and vascular tissue were very similar between the two groups. In a previous study [22] with woody species, LD was negatively related with the fraction of epidermis and mesophyll but positively related to the fraction of sclerified tissues. Villar et al. [18] found LD to be negatively related to the fraction of epidermis, as in our dataset, but in our case it explained a low proportion of LD variation (R^2^ = 0.11). Other variables related to the cell, such as the size and number of cells, could explain differences in LD. For example, the mesophyll cell size, which has been found to be negatively related with the density [22, 28]. Thus, one of the possible reasons for the lack of common patterns of LD at the tissue level is inter-specific variation at the cellular level. However, one limitation of our study is that analysis at the cellular level—that could have shed light on this assumption—was not performed, so more studies are needed to contrast these ideas.

### Relationships of leaf morphology and anatomy with chemical composition

According to our results, variation in LMA, and subsequently in LVA and LD, could have arisen from several factors which pertain to anatomical tissues and also chemical composition [18, 20]. We found different patterns for the relationships of the C and N concentrations with anatomical tissues. There were positive relationships of LMA, LVA and LD with the C concentration, but these relationships showed a phylogenetic signal (Fig 3 and S4 Table)—indicating that they pertained to certain taxonomic groups. Both the deciduous and evergreen species showed positive relationships between LMA and C, but for different reasons. While a higher LMA of deciduous species was related to an increase in C concentration in connection with the increase in VA of mesophyll tissues, for evergreen species the increase in LMA was related to the higher C concentration associated with the increase in VA of vascular plus sclerenchymatic tissues. These results suggest that, whereas deciduous species invest C in the mesophyll (photosynthetic tissues), an increase of LMA in evergreen species is the result of a greater proportion of C in structural (non-photosynthetically active) tissue.

By contrast, we found a negative relationship between LMA and N concentration, similar to other studies [1, 20, 54, 55]. The N concentration was also negatively related to LVA, LD and mesophyll VA; however, this pattern was mainly due to the differences between deciduous and evergreen species (Fig 3). These results indicate that deciduous species (short leaf life-spans) with thinner leaves have high photosynthetic rates per unit mass as a result of higher N concentrations, contrary to evergreen species [1, 54, 55]. In addition, structural differences related to high LD may be the result of a higher proportion of cell wall mass, which in fact implies a lower N concentration [56, 57]. Hence, this could explain the negative relationship between N concentration and LD for the deciduous species.

### Environmental factors and leaf structure

Different environmental conditions may impose different selective pressures on plants, driving traits to a certain degree of divergence [6]. Higher values of LMA (or lower values of SLA) contribute to long leaf life-span, nutrient retention and protection from desiccation [58, 59]. In contrast, lower values of LMA (or higher values of SLA) potentially confer an advantage in resource-uptake efficiency, by increasing the absorption surface per unit of tissue biomass [1, 12]. In this regard, our results show that the LMA distribution differed along the soil water gradient, in accordance with general ecological knowledge [8, 34, 60, 61] and our previous results—where LMA increased along a gradient of decreasing moisture [36, 59]. We have assumed that for species occurring in several sites (differing in terms of water availability), the values of a given trait as estimated in only one of the sites are representative for the whole gradient where this species can be found. Although this can modify our results, we think that it can not introduce a major bias in our conclusions, as we consider such a species in one site, connecting its leaf traits with the water content of the soil of this sampling site.

We found that the high LMA in the dry site was partly due to the leaf habit of the species present. Thus, sclerophyllous evergreen species from the driest part of the environmental gradient (such as *Quercus ilex* or *Rosmarinus officinalis*) showed higher values of LMA, while deciduous species, at humid sites, showed lower values (i.e. *Fraxinus angustifolia* or *Ulmus minor*). On the contrast, deciduous species are usually less tolerant of more-stressing conditions; thus, under strong environmental pressures (such as water scarcity), they are excluded or scarce [5, 59]. While, in more-productive (e.g. wetter) environments, the disproportional competition limits the supply of light, excluding sclerophyllous evergreen species from these wetter sites [59]. The variation of LD along the soil water gradient was not only the result of leaf habit differences, because this variation was also found within leaf habits. In this regard, the changes in LD distribution along the water gradient could be also related with the changes of leaf elasticity to the water conditions [34]. We want to highlight that the increase in LMA with the decrease of soil water availability was the result of an increase in LD, as no variation was found for LVA or the anatomical traits. This result is not necessarily contradictory: LVA and LD can respond independently to environmental and resource gradients (i.e. moisture or light) and may vary within or between species along the gradient [8]. In this sense, other studies support our results [8, 62]. Similarly, Poorter et al. [6] also found that LMA increased with water stress and that this change was due more to variation in LD than in LVA.

This decoupling of the tendencies of LD and LVA along the water gradient could have arisen because a high LVA do not necessarily require a long leaf life-span to pay back its cost of construction, while higher LD could be the result of greater foliar pay-back times—because increases in density are related with decreases in net assimilation rate [63]. From the anatomical perspective, experiments within species showed that species growing at low water availability decreased their leaf expansion rates. Thus, under such environmental conditions, the cells are smaller, with thicker walls. Moreover, they are more tightly packed, with a lower fraction of air spaces [19, 64–66]. These alterations of leaf tissues increase leaf density [34] and could be the mechanism that promotes these variations between species.

## Conclusions

Our results confirm that for woody Mediterranean plants, LMA variation was strongly coordinated by both LD and LVA, through differences in anatomical composition. For woody plants growing along a natural water gradient, we observed that the increase in LVA was mainly due to a greater VA of mesophyll, but, in evergreen species, LVA also depended slightly on the VA of other anatomical tissues. However, leaf density variation was not strongly related to variation in anatomical tissues. In addition, we also confirmed that morphological, anatomical and chemical characteristics differed between leaf habits: thus, deciduous species showed lower values of LMA, LVA and mesophyll thickness and higher leaf N concentration than evergreens. Along the water gradient studied, the environmental factors seem to exert a significant effect on the selection of species with certain leaf traits, resulting in the dominance of species with higher values of LMA and LD in the driest part of the gradient.

## Supporting Information

S1 Appendix Description of the calculation of the variance explained by the different sources (LVA and LD or anatomical tissues). (DOC)

S1 Fig Cross-section of the 34 species studied. (PDF)

S2 Fig The phylogenetic tree of the 34 species studied. (DOC)

S1 Table Species studied and areas where the samples were collected. (DOC)

S2 Table Location of the sampling sites. (DOC)

S3 Table Mean ± SD values of the leaf traits for deciduous and evergreen species. (DOC)

S4 Table Pearson correlation coefficients between leaf traits. (DOC)

S5 Table Linear regressions between leaf density (LD) and anatomical tissues. (DOC)

S6 Table Mean data of leaf traits and soil water content (SWC). (DOC)
