# Supplementary material for: Leaf Mass per Area (LMA) and Its Relationship with Leaf Structure and Anatomy in 34 Mediterranean Woody Species along a Water Availability Gradient
Source: PLoS One. 2016 Feb 11;11(2):e0148788. doi: 10.1371/journal.pone.0148788 (PMC4750855; doi:10.1371/journal.pone.0148788)
Supplement: S1 Appendix — (DOC) [file pone.0148788.s001.doc]

**S1 Appendix. Description of the calculation of the variance explained by the different sources (LVA and LD or anatomical tissues)**

According to Lepš et al. (2011), the Sum of Squares (SS) can be decomposed into the amount of variability explained by individual terms of the model (in this example, two factors: mowing and fertilisation) and the unexplained variability (error). Thus:

SS_total_ = SS_factor 1_ + SS_factor 2_ + SS_factor 1 × factor 2_ + SS_error_

**Table 1**. Table obtained from Lepš et al. (2011), where results from two-way ANOVAs analysis and sum of squares (SS) decomposition are shown.


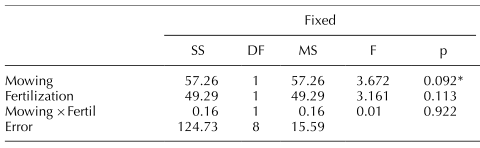


Thus, the effect of one factor without covariation is:

SS_factor 1_ = SS_total_ – (SS_error_ + SS_factor 2_ + SS_factor 1 × factor 2_)

This example is calculated for an ANOVA factorial analysis (with qualitative factors), where decomposition of the different components can be obtained in the same analysis (see Table 1). However, in the case of the multiple regressions no decomposition of the SS exists. If we consider a multiple regression with two independent variables (factor 1 and factor 2), the results of the SS is:

SS_total_ = SS_[factor 1, factor 2]_ + SS_error_

In the case of LMA (see Table 2) a multiple regression was performed with two independent variables (LVA and LD). Thus, the total of the model is explained as:

**Table 2**. Results from the multiple regression between LMA and both LVA and LD.

In order to obtain the variability explained by each independent component (LVA and LD) we could make a simple regression analysis with each component separately. However, if the two effects are positively correlated (i.e. LD and LVA), then the SS explained will be higher than when the two effects are independent. Thus, the sum of the SS explained by each component independently is higher than the SS_total_.

For example, as observed in Table 3, when some correlation exists between elements the total SS explained by the model using each factor separately (SS_LD_ + SS_LVA_ = 35014 + 28264 = 63278) is higher than the total SS explained by the two variables included in the multiple regression model (SS _[LD, LVA]_ = 53455), or even than the SS_total_ explained by the multiple regression model (55735).

-SS_LD_ refers to the SS explained with a linear regression of LMA with LD.

-SS_LVA_ refers to the SS explained with a linear regression of LMA with LVA.

-SS _[LD, LVA]_ refers to the SS explained with a linear regression of LMA with LVA.

**Table 3**. Sum of squares decomposition from the different regression analyses with LMA: **A**) multiple regression between LMA and both LD and LVA, **B**) simple regression between LMA and LVA and **C**) simple regression between LMA and LD.

In order to avoid the covariation effect and discern the explanatory percentage of each independent factor (without covariation), we should analyse each factor independently and then we can obtain the difference between the total model (SS_[LD, LVA]_ and the model explained with the other component, thus in general:

SSexp_factor 1= SSexp_[factor 1, factor 2] – SSexp_factor 2

According to this, in our example with LMA:

SS_LD_ = SS_[LD, LVA]_ – SS_LVA_; and SS_LVA_ = SS_[LD, LVA]_ – SS_LD_

Then, we can calculate the percentage of variation of LMA due to LD or LVA as:

LD (%) = 100 × [SS_LD_/ SS_total_]; LVA (%) = 100 × [SS_LVA_/ SS_total_]

**Table 4**. The variability of LMA explained by each independent factor (LD and LVA).

So, in this case, we can conclude that the variation in LMA is explained by LD (45 %) and LVA (33%).

We used the same criterion to obtain the variability explained by the different components from the LVA. First, we performed the multiple regression analysis with all the histological components (SS_LVA =_ SS_[VAepi, VAmes, VAvst, VAair]_), obtaining the total variability explained by the model. After that, we carried out multiple regressions, removing one component in each multiple regression. For example, the variability explained by the epidermis is:

SS_VAepi_ = SS_LVA_ - SS_[VAmes, VAvst, VAair]_

We proceed with the same calculations for all the tissues.

**References**

Lepš J, de Bello F, Šmilauer P, Doležal J. Community trait response to environment: disentangling species turnover vs intraspecific trait variability effects. Ecography 2011; 34: 856- 863.
