# Supplementary material for: Leaf Mass per Area (LMA) and Its Relationship with Leaf Structure and Anatomy in 34 Mediterranean Woody Species along a Water Availability Gradient
Source: PLoS One. 2016 Feb 11;11(2):e0148788. doi: 10.1371/journal.pone.0148788 (PMC4750855; doi:10.1371/journal.pone.0148788)

S1 Fig. Cross sections of the 34 species studied.

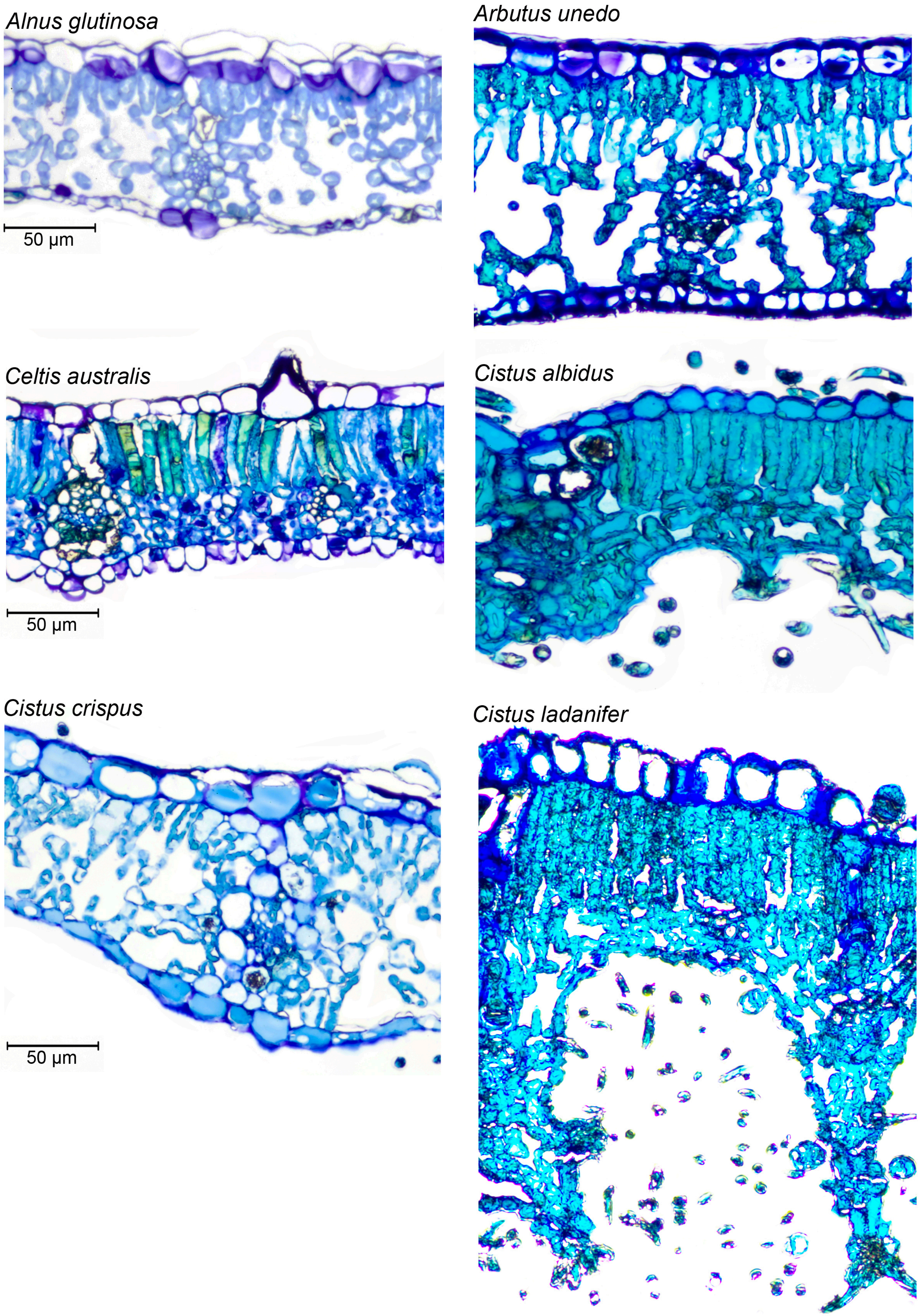
Alnus glutinosa
50 µm
Arbutus unedo
Celtis australis
50 µm
Cistus albidus
Cistus crispus
50 µm
Cistus ladanifer

Cistus monspeliensis

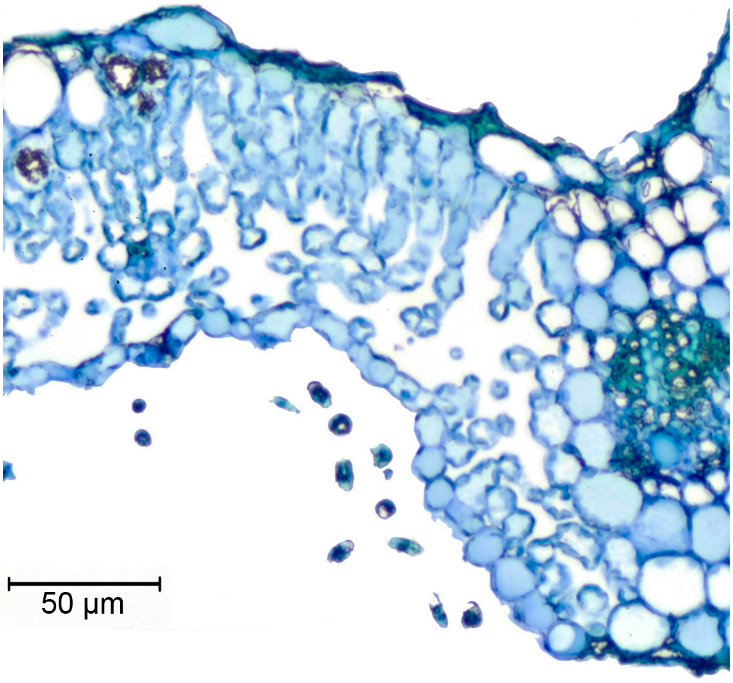

Crataegus monogyna

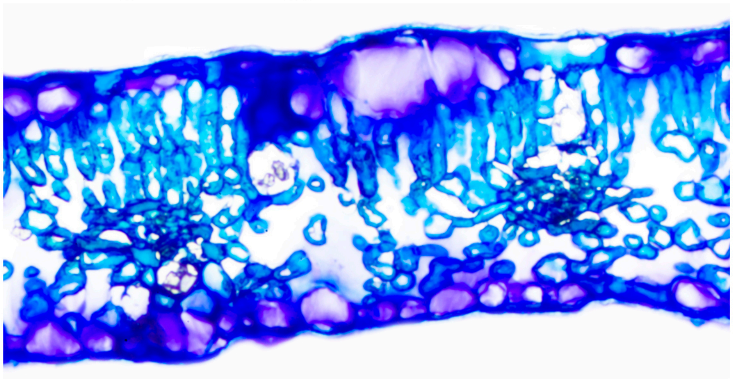

Cydonia oblonga

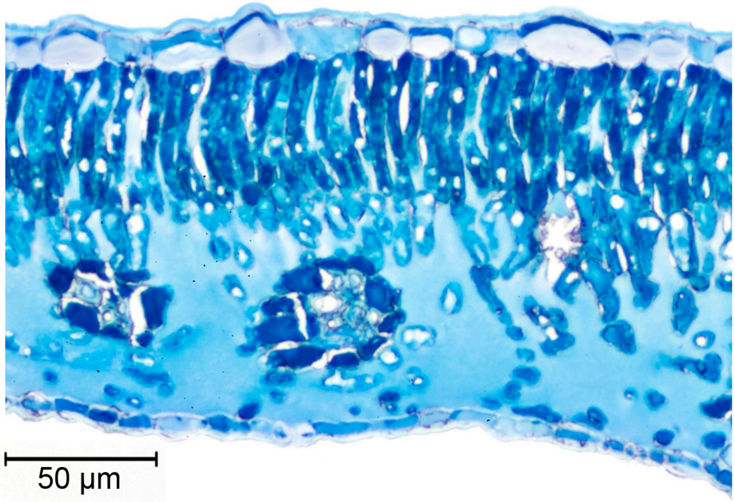

Ficus carica

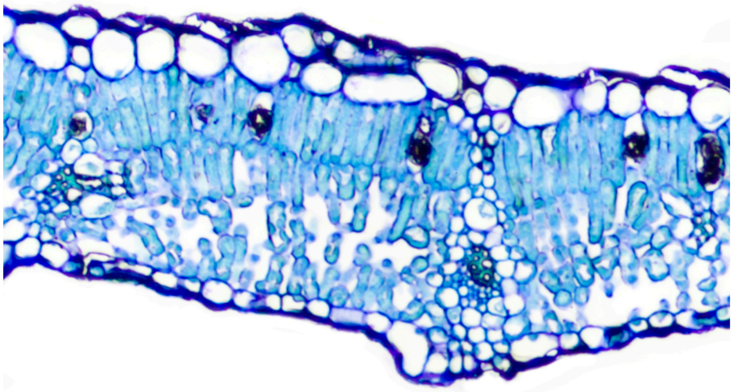

Fraxinus angustifolia

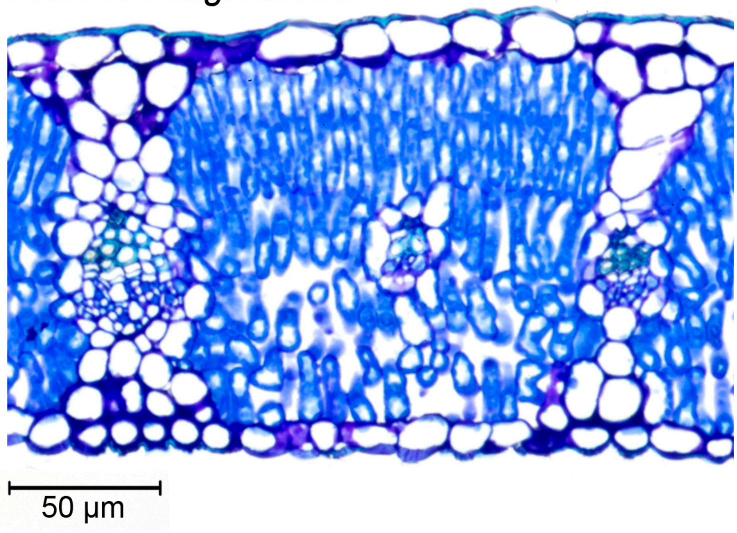

Jasminum fruticans

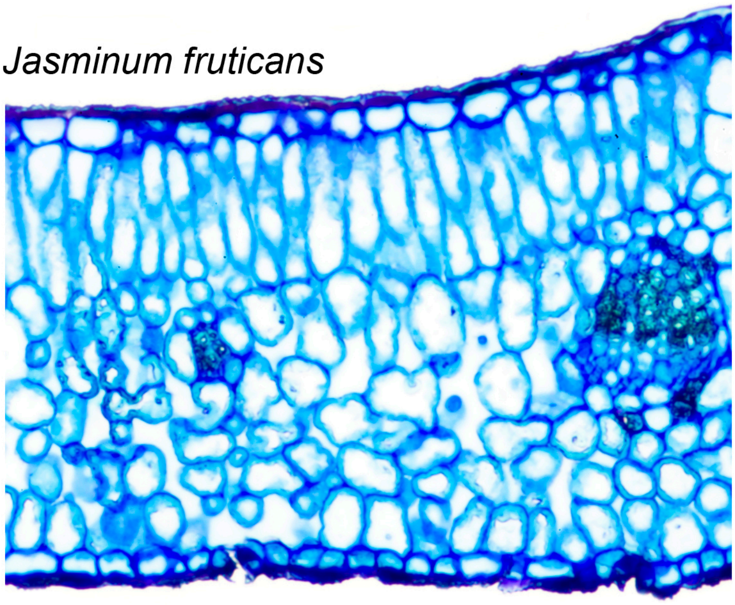

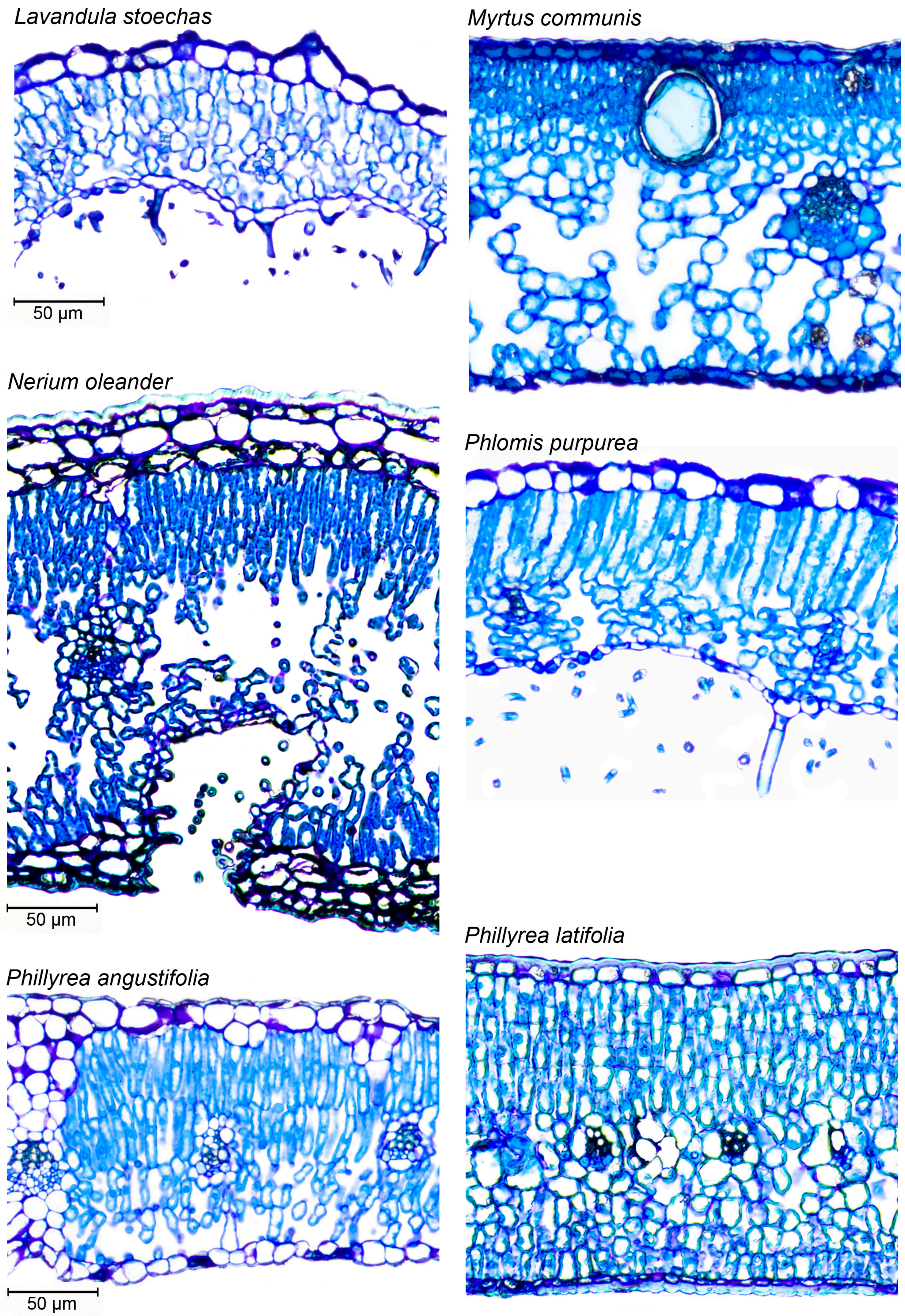
Lavandula stoechas
50 µm
Myrtus communis
Nerium oleander
50 µm
Phlomis purpurea
Phillyrea angustifolia
50 µm
Phillyrea latifolia

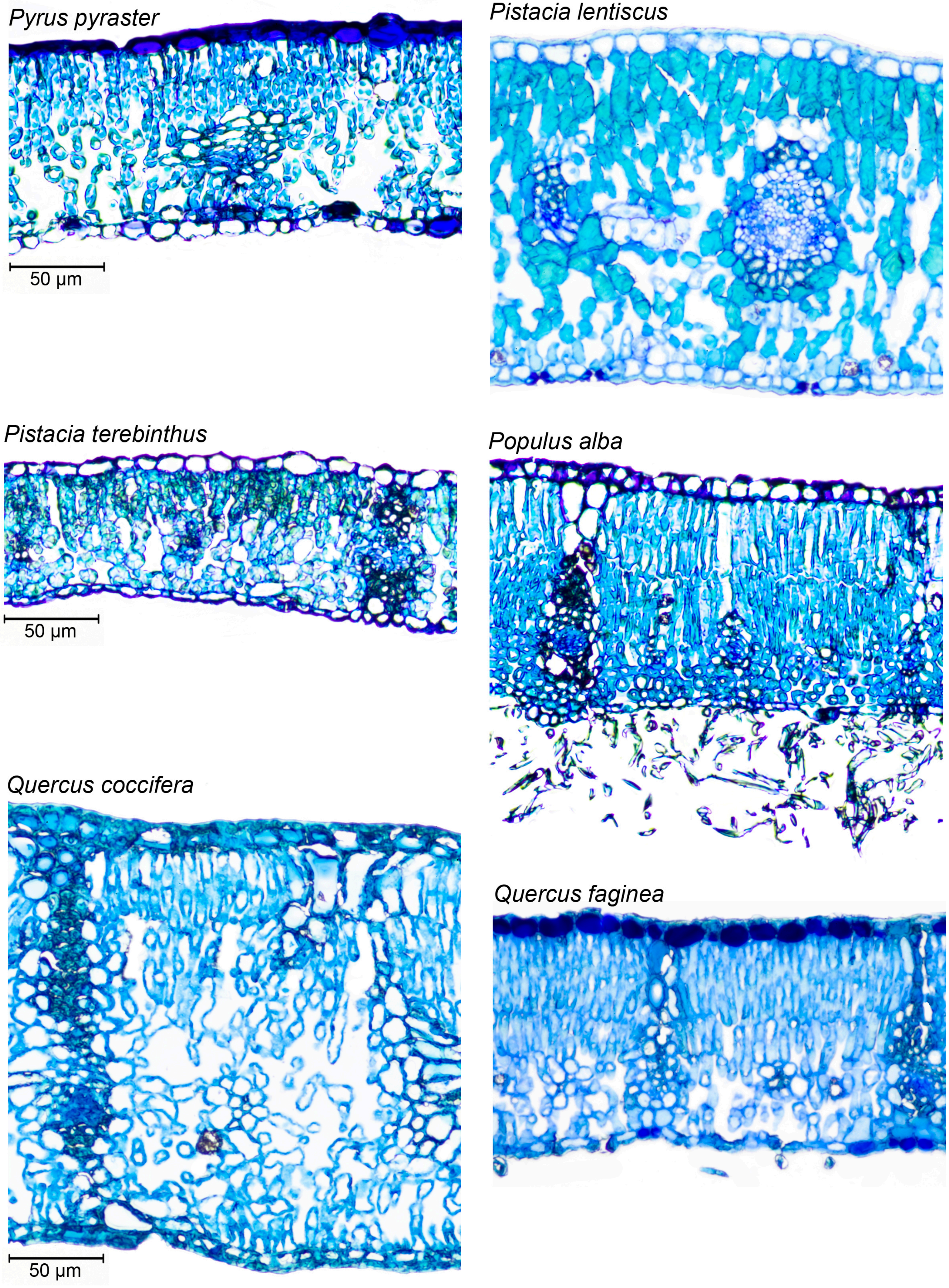
Pyrus pyraster
50 µm
Pistacia lentiscus
Pistacia terebinthus
50 µm
Populus alba
Quercus coccifera
50 µm
Quercus faginea

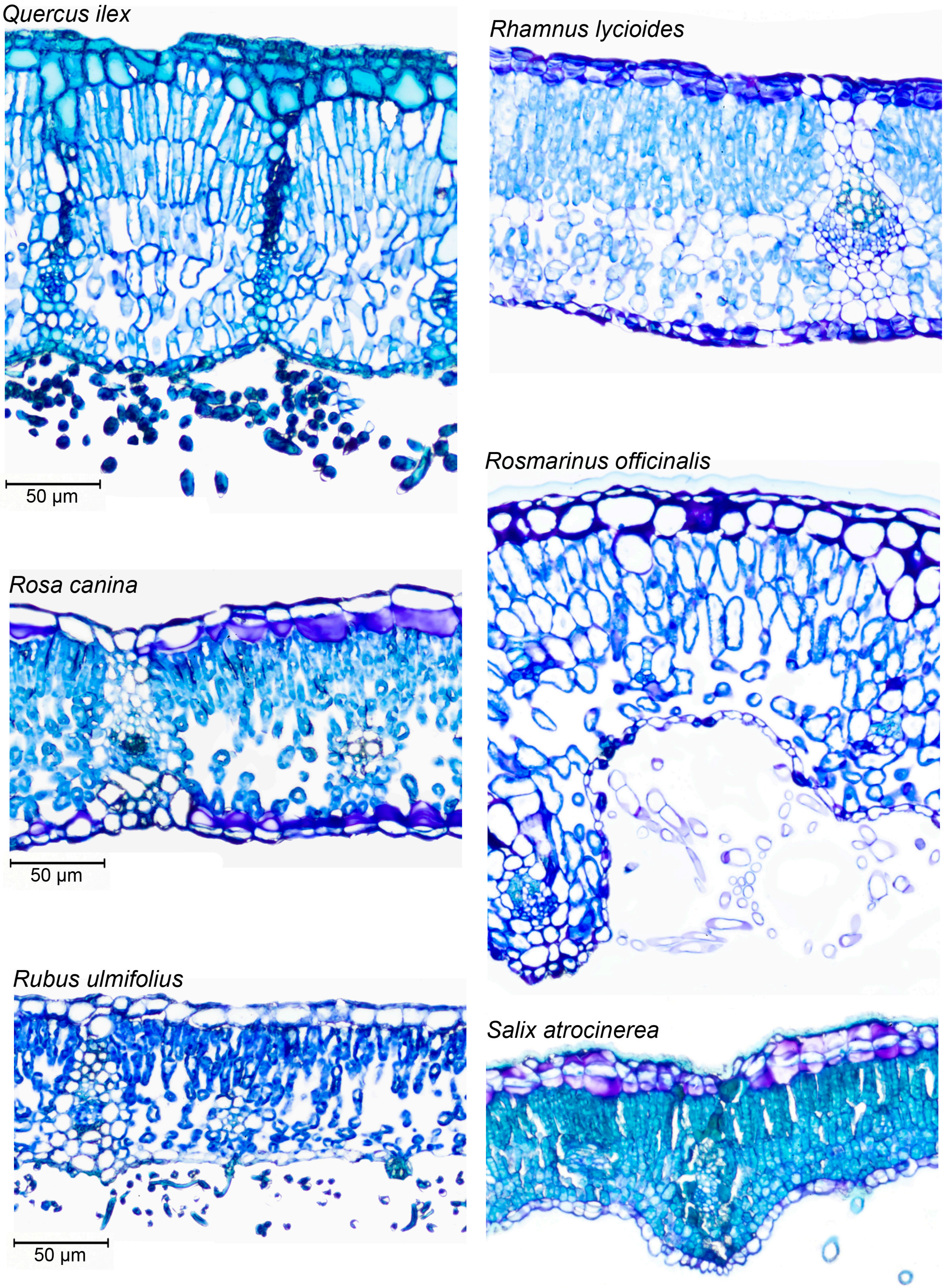
Quercus ilex
50 µm
Rhamnus lycioides
Rosmarinus officinalis
Rosa canina
50 µm
Rubus ulmifolius
50 µm
Salix atrocinerea

Smilax aspera

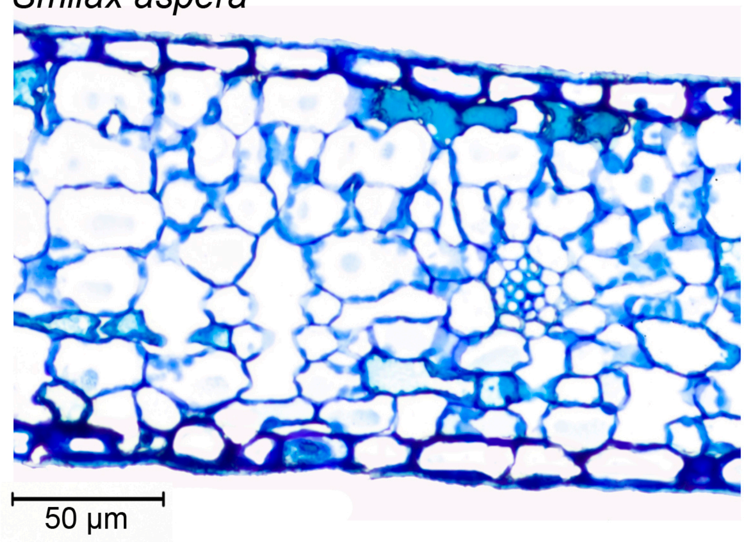

Teucrium fruticans

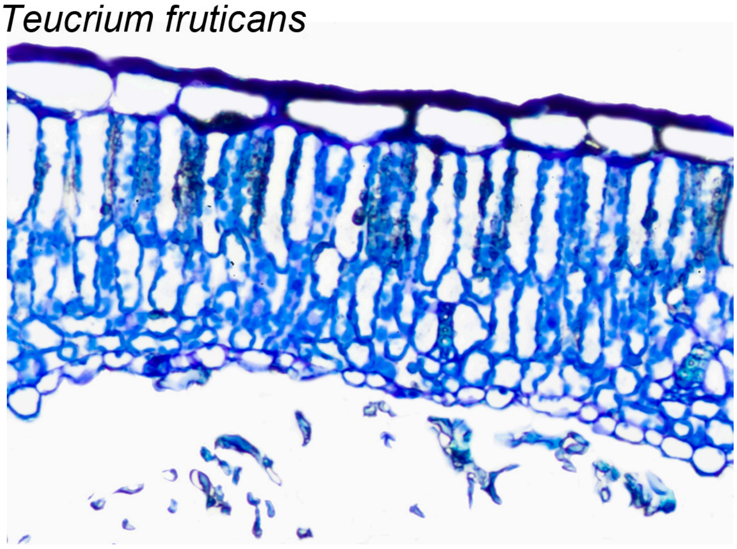

Ulmus minor

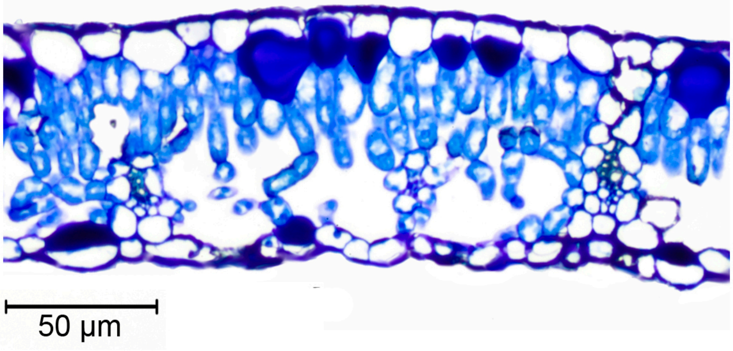

Vitis vinifera

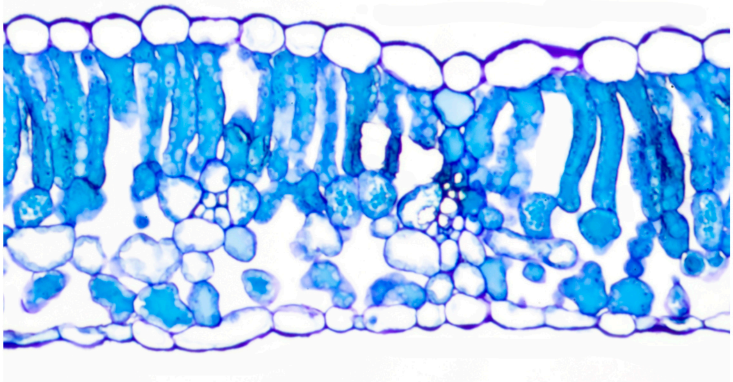

S2 Fig. The phylogenetic tree of the 34 studied species was obtained with the help of the Phylomatic program as implemented in Phylocom 4.2 and the reference phylogeny contained in R20120829.new (Webb, Ackerly & Kembel 2008).

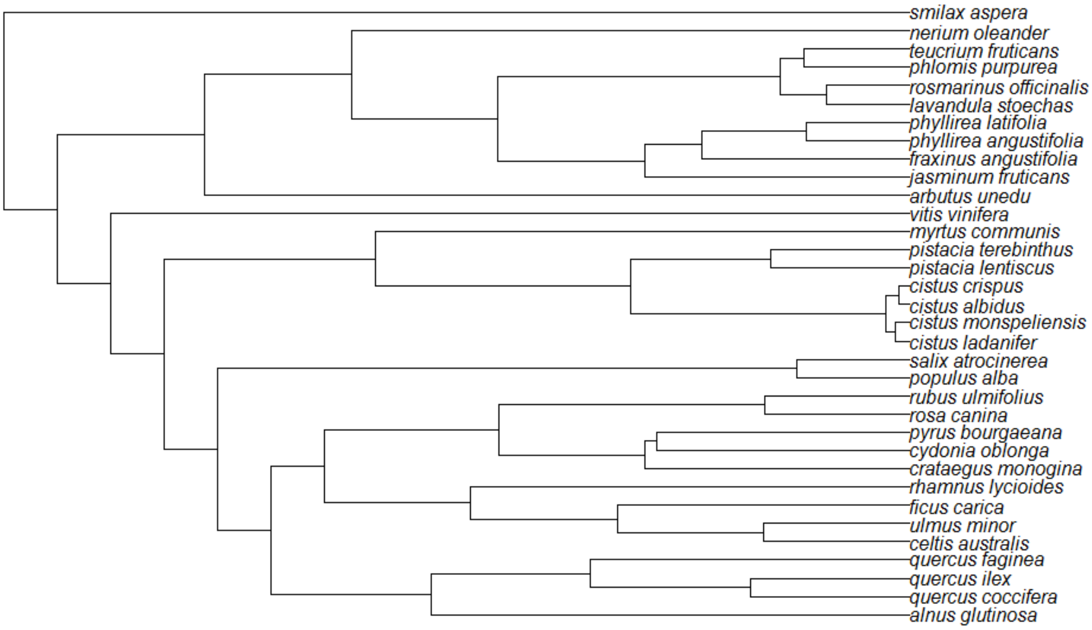

Supplement: S1 Fig — (PDF) [file pone.0148788.s002.pdf]
