# Supplementary material for: Leaf Mass per Area (LMA) and Its Relationship with Leaf Structure and Anatomy in 34 Mediterranean Woody Species along a Water Availability Gradient
Source: PLoS One. 2016 Feb 11;11(2):e0148788. doi: 10.1371/journal.pone.0148788 (PMC4750855; doi:10.1371/journal.pone.0148788)

**S2 Fig. The phylogenetic tree of the 34 studied species** was obtained with the help of the Phylomatic program as implemented in Phylocom 4.2 and the reference phylogeny contained in R20120829.new (Webb, Ackerly & Kembel 2008).


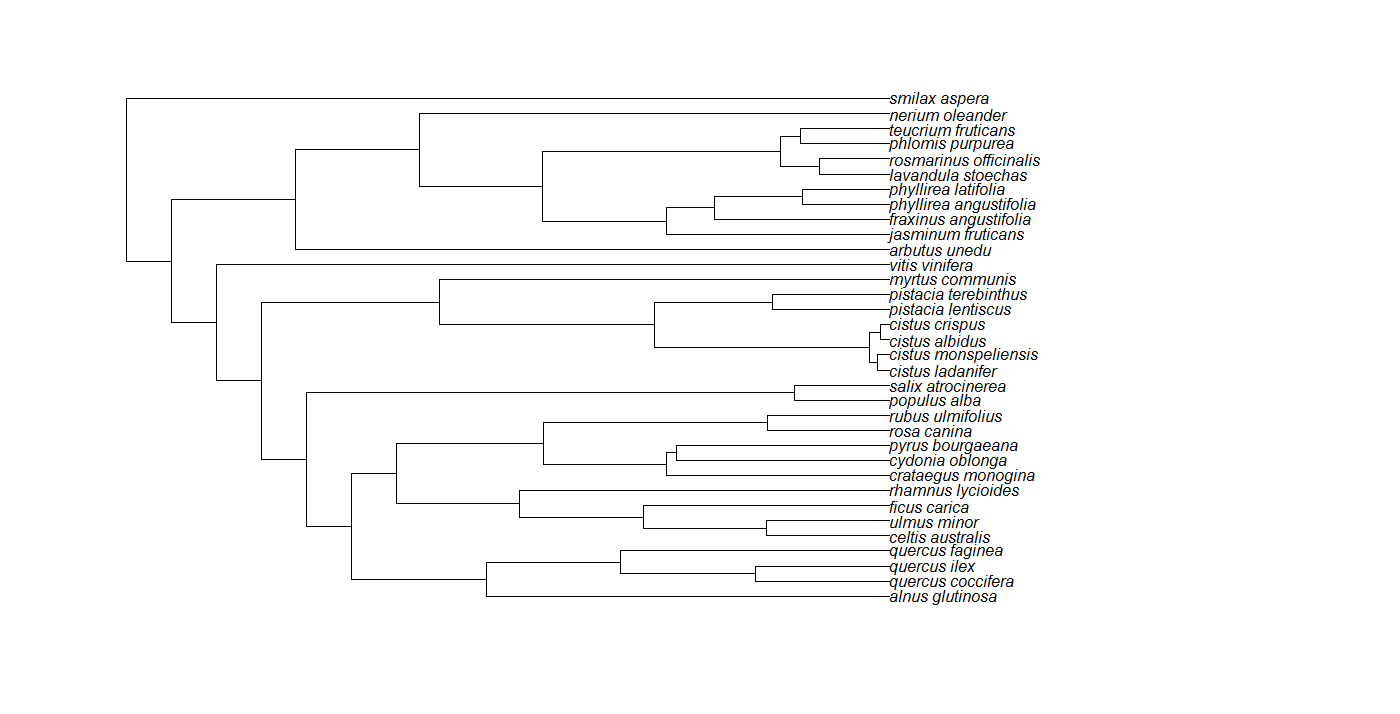

Supplement: S2 Fig — (DOC) [file pone.0148788.s003.doc]
