# Supplementary material for: Leaf Mass per Area (LMA) and Its Relationship with Leaf Structure and Anatomy in 34 Mediterranean Woody Species along a Water Availability Gradient
Source: PLoS One. 2016 Feb 11;11(2):e0148788. doi: 10.1371/journal.pone.0148788 (PMC4750855; doi:10.1371/journal.pone.0148788)
Supplement: S1 Table — (DOC) [file pone.0148788.s004.doc]

**S1 Table. Species studied and areas where the samples were collected** (see de la Riva et al 2015 for site description). T, tree; ST, small tree or arborescent shrub; S, shrub; D, deciduous; E, evergreen.

| **Species** | **Family** | **Life habit** | **Leaf habit** | **Sample site** | **Latitude** | **Longitude** |
| --- | --- | --- | --- | --- | --- | --- |
|
| *Alnus glutinosa* | Betulaceae | Tree | Winter deciduous | Bejarano river | 37° 56´ 90"N | 4° 53´ 60"W |
| *Arbutus unedo* | Ericaceae | Arborescent shrub | Evergreen | Las Tonadas high hill | 38° 1´ 32"N | 5° 1´ 65"W |
| *Celtis australis* | Cannabaceae | Tree | Winter deciduous | Pedroches stream | 37° 55´ 73"N | 4° 46´ 16"W |
| *Cistus albidus* | Cistaceae | Shrub | Evergreen | Oribe bajo high hill | 37° 56´ 95"N | 4° 46´ 22"W |
| *Cistus crispus* | Cistaceae | Shrub | Evergreen | Oribe bajo high hill | 37° 56´ 95"N | 4° 46´ 22"W |
| *Cistus ladanifer* | Cistaceae | Shrub | Evergreen | Las Tonadas high hill | 38° 1´ 32"N | 5° 1´ 65"W |
| *Cistus monspeliensis* | Cistaceae | Shrub | Evergreen | Las Tonadas medium hill | 38° 1´ 09"N | 5° 1´ 57"W |
| *Crataegus monogyna* | Rosaceae | Arborescent shrub | Winter deciduous | Pedroches stream | 37° 55´ 73"N | 4° 46´ 16"W |
| *Cydonia oblonga* | Rosaceae | Tree | Winter deciduous | Las Tonadas medium hill | 38° 1´ 09"N | 5° 1´ 57"W |
| *Ficus carica* | Moraceae | Tree | Winter deciduous | Bejarano river | 37° 56´ 90"N | 4° 53´ 60"W |
| *Fraxinus angustifolia* | Oleaceae | Tree | Winter deciduous | Orejon Stream | 38° 1´ 73"N | 5° 1´ 54"W |
| *Jasminum fruticans* | Oleaceae | Shrub | Evergreen | El Molinillo medium hill | 37° 56´ 64"N | 4° 53´ 62"W |
| *Lavandula stoechas* | Lamiaceae | Shrub | Evergreen | Oribe bajo medium hill | 37° 55´ 83"N | 4° 46´ 93"W |
| *Myrtus communis* | Mirtaceae | Shrub | Evergreen | Oribe bajo medium hill | 37° 55´ 83"N | 4° 46´ 93"W |
| *Nerium oleander* | Apocynaceae | Arborescent shrub | Evergreen | Bejarano river | 37° 56´ 90"N | 4° 53´ 60"W |
| *Phlomis purpurea* | Lamiaceae | Shrub | Evergreen | Oribe bajo high hill | 37° 56´ 95"N | 4° 46´ 22"W |
| *Phillyrea angustifolia* | Oleaceae | Arborescent shrub | Evergreen | Las Tonadas high hill | 38° 1´ 32"N | 5° 1´ 65"W |
| *Phillyrea latifolia* | Oleaceae | Arborescent shrub | Evergreen | El Molinillo medium hill | 37° 56´ 64"N | 4° 53´ 62"W |
| *Pistacia lentiscus* | Anacardiaceae | Arborescent shrub | Evergreen | Oribe bajo medium hill | 37° 55´ 83"N | 4° 46´ 93"W |
| *Pistacia terebinthus* | Anacardiaceae | Arborescent shrub | Winter deciduous | El Molinillo medium hill | 37° 56´ 64"N | 4° 53´ 62"W |
| *Populus alba* | Salicaceae | Tree | Winter deciduous | Orejon Stream | 38° 1´ 73"N | 5° 1´ 54"W |
| *Pyrus bourgaeana* | Rosaceae | Tree | Winter deciduous | Las Tonadas medium hill | 38° 1´ 09"N | 5° 1´ 57"W |
| *Quercus coccifera* | Fagaceae | Arborescent shrub | Evergreen | Oribe bajo medium hill | 37° 55´ 83"N | 4° 46´ 93"W |
| *Quercus faginea* | Fagaceae | Tree | Winter deciduous | El Molinillo medium hill | 37° 56´ 64"N | 4° 53´ 62"W |
| *Quercus ilex spp.ballota* | Fabaceae | Tree | Evergreen | Oribe bajo medium hill | 37° 55´ 83"N | 4° 46´ 93"W |
| *Rhamnus lycioides* | Rhamnaceae | Shrub | Evergreen | Pedroches stream | 37° 55´ 73"N | 4° 46´ 16"W |
| *Rosa canina* | Rosaceae | Shrub | Winter deciduous | Las Tonadas medium hill | 38° 1´ 09"N | 5° 1´ 57"W |
| *Rosmarinus officinalis* | Lamiaceae | Shrub | Evergreen | El Molinillo high hill | 37° 56´ 64"N | 4° 53´ 62"W |
| *Rubus ulmifolius* | Rosaceae | Shrub | Evergreen | Las Tonadas medium hill | 38° 1´ 09"N | 5° 1´ 57"W |
| *Salix atrocinerea* | Salicaceae | Tree | Winter deciduous | Orejon Stream | 38° 1´ 73"N | 5° 1´ 54"W |
| *Smilax aspera* | Smilacaceae | Vine | Evergreen | El Molinillo medium hill | 37° 56´ 64"N | 4° 53´ 62"W |
| *Teucrium fruticans* | Lamiaceae | Shrub | Evergreen | El Molinillo high hill | 37° 56´ 81"N | 4° 53´ 44"W |
| *Ulmus minor* | Ulmaceae | Tree | Winter deciduous | Bejarano river | 37° 56´ 90"N | 4° 53´ 60"W |
| *Vitis vinifera* | Vitaceae | Vine | Winter deciduous | Pedroches stream | 37° 55´ 73"N | 4° 46´ 16"W |
