# Supplementary material for: Leaf Mass per Area (LMA) and Its Relationship with Leaf Structure and Anatomy in 34 Mediterranean Woody Species along a Water Availability Gradient
Source: PLoS One. 2016 Feb 11;11(2):e0148788. doi: 10.1371/journal.pone.0148788 (PMC4750855; doi:10.1371/journal.pone.0148788)
Supplement: S2 Table — (DOC) [file pone.0148788.s005.doc]

**S2 Table**. **Location of the sampling sites.** The annual rainfall and the soil water content is shown. Sites are ordered by soil water content.

| **Sample site** | **Latitude** | **Longitude** | **Rainfall (mm)** | **SWS**  **(L m^-2^)** |
| --- | --- | --- | --- | --- |
|
| Las Tonadas high hill | 38° 1´ 32"N | 5° 1´ 65"W | 668 | 25.61 |
| El Molinillo high hill | 37° 56´ 64"N | 4° 53´ 62"W | 647 | 30.65 |
| El Molinillo medium hill | 37° 56´ 64"N | 4° 53´ 62"W | 647 | 49.26 |
| Las Tonadas medium hill | 38° 1´ 09"N | 5° 1´ 57"W | 668 | 50.83 |
| Oribe bajo high hill | 37° 56´ 95"N | 4° 46´ 22"W | 638 | 62.34 |
| Oribe bajo medium hill | 37° 55´ 83"N | 4° 46´ 93"W | 638 | 65.38 |
| Orejon Stream | 38° 1´ 73"N | 5° 1´ 54"W | 668 | 80.53 |
| Pedroches stream | 37° 55´ 73"N | 4° 46´ 16"W | 638 | 81.19 |
| Bejarano river | 37° 56´ 90"N | 4° 53´ 60"W | 647 | 93.63 |
