# Supplementary material for: Leaf Mass per Area (LMA) and Its Relationship with Leaf Structure and Anatomy in 34 Mediterranean Woody Species along a Water Availability Gradient
Source: PLoS One. 2016 Feb 11;11(2):e0148788. doi: 10.1371/journal.pone.0148788 (PMC4750855; doi:10.1371/journal.pone.0148788)
Supplement: S3 Table — (DOC) [file pone.0148788.s006.doc]

**S3 Table**. **Mean ± SD values of the leaf traits for deciduous and evergreens.** The data of anatomical tissues are presented in absolute values (tissue thickness) and percentage of the different tissues (i.e. tissue volumes fractions). The significance (P) of the comparison between deciduous and evergreen species following a phylogenetic generalized least squares model (PGLS) is presented. The level of significance is expressed as follows: a 0.05 ≤ P < 0.10, * *P*< 0.05, ** *P*< 0.01, *** *P*< 0.001.

|  |  | **Deciduous** | | |  | **Evergreen** | | |  | *P* |
| --- | --- | --- | --- | --- | --- | --- | --- | --- | --- | --- |
|  |  |  |  |  |  |  |  |  |  |  |
| **LMA (g m^-^²)** |  | 67.09 | ± | 27.78 |  | 102.90 | ± | 43.00 |  | ** |
| **LVA (mL m^-2^)** |  | 132.08 | ± | 27.26 |  | 180.16 | ± | 56.36 |  | ** |
| **LD (g mL^-1^)** |  | 0.49 | ± | 0.15 |  | 0.57 | ± | 0.17 |  | ns |
|  |  |  |  |  |  |  |  |  |  |  |
| **Epidermis VA (mL m^-2^)** |  | 28.56 | ± | 6.29 |  | 32.16 | ± | 17.45 |  | ns |
| **Mesophyll VA (mL m^-2^)** |  | 69.78 | ± | 23.91 |  | 103.10 | ± | 31.33 |  | *** |
| **Air spaces VA (mL m^-2^)** |  | 19.50 | ± | 5.25 |  | 24.74 | ± | 12.68 |  | ns |
| **Vas+ Scl VA (mL m^-2^)** |  | 13.68 | ± | 7.69 |  | 18.99 | ± | 17.48 |  | ns |
|  |  |  |  |  |  |  |  |  |  |  |
| **Mesophyll (%)** |  | 51.30 | ± | 8.97 |  | 57.80 | ± | 9.88 |  | * |
| **Epidermis (%)** |  | 22.52 | ± | 5.49 |  | 18.20 | ± | 5.70 |  | * |
| **Air spaces (%)** |  | 10.86 | ± | 5.66 |  | 10.05 | ± | 4.64 |  | ns |
| **Vas + Scl (%)** |  | 14.85 | ± | 6.33 |  | 13.28 | ± | 7.94 |  | ns |
|  |  |  |  |  |  |  |  |  |  |  |
| **LNC (%)** |  | 2.59 | ± | 0.52 |  | 1.91 | ± | 0.48 |  | *** |
| **LCC (%)** |  | 47.30 | ± | 3.36 |  | 48.97 | ± | 1.80 |  | a |
