# Supplementary material for: Leaf Mass per Area (LMA) and Its Relationship with Leaf Structure and Anatomy in 34 Mediterranean Woody Species along a Water Availability Gradient
Source: PLoS One. 2016 Feb 11;11(2):e0148788. doi: 10.1371/journal.pone.0148788 (PMC4750855; doi:10.1371/journal.pone.0148788)
Supplement: S4 Table — (DOC) [file pone.0148788.s007.doc]

**S4 Table. Pearson correlation coefficients between leaf traits** for: **A**) all species data (above diagonal) and phylogenetically independent contrasts (below diagonal) and **B**) for deciduous species data (above diagonal) and for evergreen species (below diagonal). The level of significance is expressed as follows: * *P*< 0.05, ** *P*< 0.01, *** *P*< 0.001.

|  |  |  |  |  |  |  |  |  |  |
| --- | --- | --- | --- | --- | --- | --- | --- | --- | --- |
| **A)** | **LMA** | **LVA** | **LD** | **Epi VA** | **Mes VA** | **Vas + Scl VA** | **Air VA** | **LCC** | **LNC** |
| **LMA** |  | 0.71*** | 0.79*** | 0.28 | 0.64*** | 0.27 | 0.63*** | 0.50** | -0.49** |
| **LVA** | 0.33* |  | 0.18 | 0.63** | 0.84*** | 0.59** | 0.62** | 0.38* | -0.47** |
| **LD** | 0.91*** | 0.22 |  | -0.07 | 0.22 | 0.36* | -0.15 | 0.43** | -0.36* |
| **Epi VA** | 0.69*** | 0.33* | 0.51** |  | 0.27 | 0.37* | 0.46** | 0.01 | -0.20 |
| **Mes VA** | 0.67*** | 0.37* | 0.41* | 0.46 |  | 0.24 | 0.33 | 0.38* | -0.42** |
| **Vas + Scl VA** | 0.77*** | 0.57*** | 0.66*** | 0.60*** | 0.37* |  | 0.29 | 0.35* | -0.31 |
| **Air VA** | -0.48** | -0.04 | -0.65*** | -0.11 | -0.15 | 0.29 |  | 0.18 | -0.28 |
| **LCC** | 0.12 | 0.49** | 0.09 | -0.08 | 0.16 | 0.35* | 0.12 |  | -0.50** |
| **LNC** | -0.58*** | -0.38* | -0.43* | -0.43* | -0.49** | -0.49** | 0.24 | -0.50** |  |
|  |  |  |  |  |  |  |  |  |  |
|  |  |  |  |  |  |  |  |  |  |
| **B)** | **LMA** | **LVA** | **LD** | **Epi VA** | **Mes VA** | **Vas + Scl VA** | **Air VA** | **LCC** | **LNC** |
| **LMA** |  | 0.79** | 0.91*** | -0.01 | 0.80** | 0.53* | -0.32 | 0.62* | -0.49 |
| **LVA** | 0.61** |  | 0.48 | 0.23 | 0.95*** | 0.57* | -0.23 | 0.55* | -0.35 |
| **LD** | 0.75*** | -0.02 |  | -0.09 | 0.51 | 0.39 | -0.27 | 0.51 | -0.55* |
| **Epi VA** | 0.31 | 0.71*** | -0.12 |  | 0.04 | -0.28 | 0.29 | -0.05 | -0.46 |
| **Mes VA** | 0.46* | 0.77*** | -0.06 | 0.29 |  | 0.50 | -0.41 | 0.62* | -0.29 |
| **Vas + Scl VA** | 0.65** | 0.58** | 0.34 | 0.43 | 0.11 |  | -0.43 | -0.01 | -0.01 |
| **Air VA** | 0.27 | 0.711*** | -0.22 | 0.47* | 0.39 | 0.34 |  | 0.11 | 0.03 |
| **LCC** | 0.38 | 0.21 | 0.31 | 0.01 | -0.01 | 0.62** | 0.14 |  | -0.44 |
| **LNC** | -0.28 | -0.28 | -0.11 | -0.11 | -0.14 | -0.36 | -0.25 | -0.41 |  |
