# Supplementary material for: Leaf Mass per Area (LMA) and Its Relationship with Leaf Structure and Anatomy in 34 Mediterranean Woody Species along a Water Availability Gradient
Source: PLoS One. 2016 Feb 11;11(2):e0148788. doi: 10.1371/journal.pone.0148788 (PMC4750855; doi:10.1371/journal.pone.0148788)
Supplement: S5 Table — (DOC) [file pone.0148788.s008.doc]

**S5 Table. Linear regressions between leaf density (LD) with anatomical tissues**, expressed as tissue volume fractions). The level of significance is expressed as follows: a 0.05 ≤ *P* < 0.10, * *P*< 0.05.

|  | **LD** | | | | | | | |
| --- | --- | --- | --- | --- | --- | --- | --- | --- |
|  | **All** | |  | **Deciduous** | |  | **Evergreen** | |
|  | Rel | R² |  | Rel | R² |  | Rel | R² |
| **Epidermis (%)** | - | 0.11 * |  | ns | |  |  | ns |
|  |  |  |  |  |  |  |  |  |
| **Mesophyll (%)** |  | ns |  | + | 0.24 a |  |  | ns |
|  |  |  |  |  |  |  |  |  |
| **Vas + Scl (%)** |  | ns |  | ns | |  | + | 0.16 a |
|  |  |  |  |  |  |  |  |  |
| **Air spaces (%)** | - | 0.11 * |  | ns | |  |  | ns |
|  |  |  |  |  |  |  |  |  |
