# Supplementary material for: Leaf Mass per Area (LMA) and Its Relationship with Leaf Structure and Anatomy in 34 Mediterranean Woody Species along a Water Availability Gradient
Source: PLoS One. 2016 Feb 11;11(2):e0148788. doi: 10.1371/journal.pone.0148788 (PMC4750855; doi:10.1371/journal.pone.0148788)
Supplement: S6 Table — (DOC) [file pone.0148788.s009.doc]

**S6 Table. Mean data of leaf traits and soil water content (SWC)** of the sites where species were collected.

| **Species** | **LMA (g m^-^²)** | **LVA (mL m^-2^)** | **LD**  **(g mL^-1^)** | **Epidermis VA (mL m^-2^)** | **Mesophyll VA (mL m^-2^)** | **Air spaces VA (mL m^-2^)** | **Vas+ Scl VA (mL m^-2^)** | **LN (%)** | **LC (%)** | **SWC**  **(g m^-2^)** |
| --- | --- | --- | --- | --- | --- | --- | --- | --- | --- | --- |
| *Alnus glutinosa* | 27.8 | 89.1 | 0.31 | 23.1 | 34.4 | 24.0 | 7.4 | 3.46 | 48.9 | 93.63 |
| *Arbutus unedo* | 120.3 | 219.5 | 0.55 | 47.8 | 95.5 | 42.8 | 33.4 | 1.63 | 50.0 | 25.61 |
| *Celtis australis* | 56.8 | 118.7 | 0.48 | 30.9 | 61.6 | 11.5 | 12.3 | 3.03 | 44.8 | 81.19 |
| *Cistus albidus* | 92.4 | 130.3 | 0.71 | 23.4 | 74.5 | 10.4 | 17.8 | 1.97 | 49.2 | 62.34 |
| *Cistus crispus* | 86.6 | 142.7 | 0.61 | 19.5 | 92.9 | 14.9 | 14.2 | 1.90 | 49.2 | 62.34 |
| *Cistus ladanifer* | 207.7 | 214.9 | 0.97 | 46.0 | 121.5 | 6.7 | 40.7 | 1.51 | 48.8 | 25.61 |
| *Cistus monspeliensis* | 86.1 | 160.5 | 0.54 | 31.8 | 86.1 | 21.3 | 19.6 | 2.14 | 49.2 | 50.83 |
| *Crataegus monogyna* | 73.9 | 130.1 | 0.57 | 41.4 | 58.0 | 19.9 | 10.7 | 1.61 | 49.1 | 81.19 |
| *Cydonia oblonga* | 88.0 | 150.8 | 0.58 | 28.5 | 84.1 | 12.8 | 25.4 | 2.30 | 49.4 | 50.83 |
| *Ficus carica* | 31.0 | 80.4 | 0.38 | 20.2 | 28.8 | 6.0 | 24.7 | 3.25 | 39.5 | 93.63 |
| *Fraxinus angustifolia* | 70.6 | 168.7 | 0.42 | 28.0 | 93.6 | 15.1 | 32.0 | 2.83 | 46.9 | 80.53 |
| *Jasminum fruticans* | 78.8 | 197.9 | 0.40 | 29.8 | 152.8 | 9.7 | 5.6 | 1.91 | 46.7 | 49.26 |
| *Lavandula stoechas* | 31.0 | 74.8 | 0.42 | 23.0 | 38.2 | 8.5 | 5.1 | 1.43 | 46.7 | 65.38 |
| *Myrtus communis* | 91.8 | 228.7 | 0.40 | 23.9 | 140.6 | 36.8 | 18.7 | 1.47 | 46.8 | 65.38 |
| *Nerium oleander* | 122.7 | 328.8 | 0.37 | 99.4 | 132.7 | 38.5 | 55.2 | 1.70 | 49.2 | 93.63 |
| *Phlomis purpurea* | 110.0 | 136.1 | 0.81 | 29.3 | 85.2 | 11.2 | 10.4 | 2.53 | 47.3 | 62.34 |
| *Phillyrea angustifolia* | 143.8 | 179.6 | 0.80 | 30.6 | 95.8 | 20.7 | 32.5 | 1.95 | 51.1 | 25.61 |
| *Phillyrea latifolia* | 107.6 | 191.5 | 0.56 | 23.4 | 154.7 | 7.1 | 6.3 | 1.66 | 50.3 | 49.26 |
| *Pistacia lentiscus* | 130.7 | 228.2 | 0.57 | 33.8 | 133.2 | 43.4 | 17.9 | 1.53 | 49.9 | 65.38 |
| *Pistacia terebinthus* | 85.5 | 115.2 | 0.74 | 24.0 | 59.1 | 14.2 | 17.2 | 1.85 | 48.7 | 49.26 |
| *Populus alba* | 109.9 | 170.3 | 0.65 | 25.1 | 120.0 | 3.2 | 20.7 | 2.59 | 51.8 | 80.53 |
| *Pyrus bourgaeana* | 86.3 | 152.0 | 0.57 | 29.5 | 85.9 | 15.8 | 20.8 | 2.75 | 49.8 | 50.83 |
| *Quercus coccifera* | 121.1 | 229.1 | 0.53 | 26.0 | 126.5 | 32.0 | 43.9 | 1.70 | 50.0 | 65.38 |
| *Quercus faginea* | 99.1 | 148.4 | 0.67 | 23.0 | 78.6 | 15.5 | 30.4 | 2.87 | 47.0 | 65.38 |
| *Quercus ilex spp.ballota* | 187.8 | 227.8 | 0.82 | 24.9 | 117.5 | 21.0 | 63.5 | 1.99 | 50.6 | 65.38 |
| *Rhamnus lycioides* | 45.0 | 128.3 | 0.35 | 27.2 | 84.4 | 9.8 | 6.9 | 3.29 | 47.3 | 81.19 |
| *Rosa canina* | 69.6 | 141.4 | 0.49 | 35.1 | 74.1 | 10.7 | 21.6 | 2.19 | 45.6 | 50.83 |
| *Rosmarinus officinalis* | 72.6 | 142.6 | 0.51 | 20.2 | 66.9 | 9.2 | 46.4 | 1.28 | 53.9 | 30.65 |
| *Rubus ulmifolius* | 72.4 | 125.0 | 0.58 | 23.6 | 69.3 | 8.8 | 22.3 | 2.11 | 47.8 | 50.83 |
| *Salix atrocinerea* | 81.3 | 149.3 | 0.54 | 29.0 | 83.6 | 11.4 | 25.4 | 2.12 | 50.3 | 80.53 |
| *Smilax aspera* | 68.1 | 171.6 | 0.40 | 30.8 | 112.6 | 17.9 | 8.7 | 2.72 | 47.2 | 49.26 |
| *Teucrium fruticans* | 81.5 | 145.3 | 0.56 | 29.0 | 81.1 | 9.3 | 25.9 | 1.69 | 48.2 | 30.65 |
| *Ulmus minor* | 28.6 | 121.8 | 0.23 | 39.0 | 52.5 | 17.6 | 12.7 | 2.86 | 42.0 | 93.63 |
| *Vitis vinifera* | 30.7 | 112.9 | 0.27 | 23.2 | 62.7 | 13.9 | 11.7 | 2.60 | 48.4 | 81.19 |
